# Transcriptome Analysis of Liangshan Pig Muscle Development at the Growth Curve Inflection Point and Asymptotic Stages Using Digital Gene Expression Profiling

**DOI:** 10.1371/journal.pone.0135978

**Published:** 2015-08-20

**Authors:** Linyuan Shen, Jia Luo, Jingjing Du, Chendong Liu, Xiaoqian Wu, Qiang Pu, Yuhua Fu, Qianzi Tang, Yuanrui Liu, Qiang Li, Runlin Yang, Xuewei Li, Guoqing Tang, Yanzhi Jiang, Mingzhou Li, Shunhua Zhang, Li Zhu

**Affiliations:** 1 College of Animal Science and Technology, Sichuan Agricultural University, Chengdu, Sichuan, China; 2 Key Laboratory of Agricultural Animal Genetics, Breeding, and Reproduction of Ministry of Education & Key Laboratory of Swine Genetics, Huazhong Agricultural University, Wuhan, China; 3 Mabian Gold Liangshan Agricultural Development *Co*., *Ltd*, Sichuan, China; 4 Department of Biology, College of Life and Science, Sichuan Agricultural University, Chengdu, Sichuan, China; 5 Sichuan Province General Station of Animal Husbandry, Chengdu, Sichuan, China; 6 LC Sciences, Hangzhou, Zhejiang, China; Wageningen UR Livestock Research, NETHERLANDS

## Abstract

Animal growth curves can provide essential information for animal breeders to optimize feeding and management strategies. However, the genetic mechanism underlying the phenotypic differentiation between the inflection point and asymptotic stages of the growth curve is not well characterized. Here, we employed Liangshan pigs in stages of growth at the inflection point (under inflection point: UIP) and the two asymptotic stages (before the inflection point: BIP, after the inflection point: AIP) as models to survey global gene expression in the *longissimus dorsi* muscle using digital gene expression (DGE) tag profiling. We found Liangshan pigs reached maximum growth rate (UIP) at 163.6 days of age and a weight of 134.6 kg. The DGE libraries generated 117 million reads of 5.89 gigabases in length. 21,331, 20,996 and 20,139 expressed transcripts were identified BIP, UIP and AIP, respectively. Among them, we identified 757 differentially expressed genes (DEGs) between BIP and UIP, and 271 DEGs between AIP and UIP. An enrichment analysis of DEGs proved the immune system was strengthened in the AIP stage. Energy metabolism rate, global transcriptional activity and bone development intensity were highest UIP. Meat from Liangshan pigs had the highest intramuscular fat content and most favorable fatty acid composition in the AIP. Three hundred eighty (27.70%) specific expression genes were highly enriched in QTL regions for growth and meat quality traits. This study completed a comprehensive analysis of diverse genetic mechanisms underlying the inflection point and asymptotic stages of growth. Our findings will serve as an important resource in the understanding of animal growth and development in indigenous pig breeds.

## Introduction

China possesses more than one-third, about 100, of the diverse pig breeds distributed globally [1]. However, many native Chinese pig breeds are on the edge of extinction due to a lack of optimum feeding and management strategies and the threat posed by the popularity of modern commercial foreign breeds. Pig growth curve models can be utilized to identify the nutrient requirements for growth, indicate individual-environment interactions, predict market weight and maximize return over feed costs. This information can be used by animal breeders to develop optimal feeding and management strategies [2–4]. The classical growth development model is an asymmetric, sigmoidal curve with an asymptote and one inflection point. The postnatal growth rate continues increasing slowly until it reaches a maximum at the inflection point, then decreases asymptotically [5]. Many functions including Gompertz, logistic, Bridges, and Bertalanffy have been optimized to fit the growth curve of animals [4, 6–8].

Although many studies have characterized growth curve models for different pig breeds, almost all focus solely on comparing the degree of fit of different models and identifying the inflection point [7–9]. However, the genetic mechanisms underlying the phenotypic differentiation between the inflection point and asymptotic stages of the growth curve are unclear. At present, next generation sequencing is being applied to analyze global changes in the mRNA and microRNA transcriptome during pig growth and development. A study by Zhao *et al*. compared the diversity of the mRNA transcriptome in obese (Lantang) and lean (Landrace) muscle from 35 days post-coitus to 180 days postnatal, and observed that myogenesis was almost complete before 77 days post-coitus [10]. Xu *et al*. reported the mRNA transcriptome of Meishan pig *longissimus dorsi* muscle at 65 days post conception, and 3, 60 and 120 days after birth [11]. McDaneld *et al*. compared the microRNA transcriptome of *longissimus dorsi* and *biceps femoris* muscles in pigs at three stages of fetal growth, day-old neonate and the adult [12]. There are several studies focused on muscle transcriptome diversity between different development stages [13–15], but no study has compared differences in the muscle transcriptome between the inflection point and asymptotic stages.

Liangshan is one of the native mountain-type pig breeds in China, which is known for its excellent meat quality attributes [16]. To describe the growth characteristics of Liangshan pigs, we modeled the growth curve of Liangshan pigs and estimated the maximum grow rate (occurring at the inflection point). Subsequently, we performed a comprehensive survey of the global gene expression changes in *longissimus dorsi* muscle by DGE at three developmental stages: at the inflection point (UIP), before the inflection point (BIP) and after the inflection point (AIP). The aim of our study is to identify differentially expressed genes that are linked to phenotypic differentiation (eg. grow rate, meat quality) between the inflection point and asymptotic stages. We envision that this study will serve as a valuable resource in muscle development studies and promote the pig as a model organism for the research of animal growth and development.

## Materials and Methods

### Ethics statement

All the experimental procedures and sample collection were approved by the Institutional Animal Care and Use Committee of the College of Animal Science and Technology of Sichuan Agricultural University, Sichuan, China, under permit No. DKY-B20131403 (Ministry of Science and Technology, China, revised in June 2004).

### Animal materials and tissue collection

A total of 275 female Liangshan pigs were raised from birth to 250 day old to fit the growth curve. The data of growth traits (feed conversion rate, daily feed intake and average daily gain) at 20 time points was measured to fit the growth curve by three non-linear models. Another 9 female pigs at inflection point (UIP), before inflection point (BIP) and after inflection point (AIP) were used to harvest *longissimus dorsi* muscle for transcriptome analysis, 3 pigs for each time point. The samples were rapidly separated from each carcass, immediately frozen in liquid nitrogen, and stored at −80°C until RNA and DNA extraction. All the pigs at the same age were reared in the same environment and fed the same diet *ad libitum*. The feed formulas meet or exceed the National Research Council (NRC, 1998) recommendations for the different growth stages.

### Growth curve models

Three sigmoid growth functions were involved in our study. The equation of logistic growth curve model is W_t_ = A/(1 + B*e* ^−*kt*^)[17]. The second Gompertz growth curve model is defined by the equation W_t_ = A*e* ^−*Be*^−*kt*^^^[^ ^17^ ^]^. The third growth curve model is the Von Bertalanffy[18], which is defined by the equation W_t_ = A × (1 − B*e* ^−*kt*^)^3^. In these three models, lnB/k, lnB/k and ln2B/k represent the inflection point age, respectively; A/2, A/e, and 8A/27 represent the inflection point weight, respectively; kw/2, kw, and 3kw/2 represent the maximum daily gain, respectively. Here, W_t_ is the weight where the time point (t) was recorded, A is the maximum size, k may be interpreted as the inherent relative growth rate at the start, and B is the growth curve line constant. Degree of fitting (R^2^) is used to judge the merits of the fitting model, the equation is R^2^ = 1− $\frac{\text{RSE}}{\text{RST}}$, where R^2^ is the degree of fitting, RSE is the residual sum of squares, and RST is the sum of squares of deviations.

### Phenotypic traits measurement

30 pigs were slaughtered to determine the carcass composition traits according to the methods described by Xiao *et al* [19]. All meat quality traits measurement were conducted by the methods described by Shen *et al* [16]. Fatty acid composition was analyzed by gas chromatography (Agilent 6820, Agilent Technologies, USA), free amino acid composition was analyzed by Automatic Amino Acid Analyzer (L-8800 Hitachi, Tokyo, Japan), the operating steps were in accordance with the method described by Yang *et al* [20] and Jiang *et al* [21].

### Total RNA and DNA extraction

Total RNA was extracted from *longissimus dorsi* using TRIzol (Invitrogen, CA, USA) and further purified with RNeasy column (Qiagen, USA) according to the manufacturer’s protocol. RNA integrity and concentration were analysis with the Bioanalyzer 2100 (Agilent Technologies). DNA was isolated from *longissimus dorsi* for the measurement of mtDNA copy number by using the DNeasy Blood & Tissue Kit (Qiagen, USA).

### Measurement of mitochondrial DNA (mtDNA) copy number

The relative mtDNA copy number was determined by Q-PCR. The ratio of mitochondrial genes (*ATP6*, *COX1* and *ND1*) to nuclear DNA single copy gene (*GCG*) within the same samples was used to calculate the mtDNA content (primer in S9 Table) [22]. All reactions were performed in triplicates. Relative mtDNA copy number per diploid cell = 2^△Ct^, data are expressed as mean ± SD.

### mRNA library construction and sequencing

Approximately 10 ug of total RNA representing a muscle tissue was subjected to isolate Poly (A) mRNA with poly-T oligo attached magnetic beads (Thermo-Fisher). Following purification, the mRNA was fragmented into small pieces using divalent cations under elevated temperature. Then the cleaved RNA fragments were constructed into the final cDNA library in accordance with the protocol for the Illumina RNA ligation based method (Illumina, San Diego, USA). In brief, the fragmented RNA has been dephosphorylated at the 3' end by the phosphatase and been phosphorylated at the 5' end by the PNK. The treated RNA has been cleaned up with the RNeasy MinElute Kit (Qiagen) following the instructions of the manufacturer. The purified RNA was ligated with a pre-adenylated 3' adapter, which enables the subsequent ligation of the 5' adapter. Based on the adapter sequence, a reverse transcription followed by PCR used to create cDNA constructs. The average insert size for the single-end libraries was 300 bp (±50 bp). And then we performed the single end sequencing (50 bp) on an Illumina Hiseq2000 at the (LC Sciences,USA) following the vendor's recommended protocol.

### Transcriptome data analysis

The raw data containing adaptor sequences, reads with low quality sequences and unknown nucleotides N were filtered to obtain clean reads with 50 nt in length. The raw datasets have been submitted to NCBI Gene Expression Omnibus database with the accession number GSE69113. Clean reads were then conducted for quality assessment. These include classification of total and distinct reads and show their percentage in the library, analyze saturation of the library and correlation analysis of biological replicates. All clean reads were mapped to the transcripts sequence of by bowtie (1.0.0), only 1 bp mismatch is allowed. For monitoring the mapping events on both strands, both of the sense and the complementary antisense sequences were included in the data collection. The number of perfect clean reads corresponding to each gene was calculated and normalized to the number of Reads Per Kilobase of exon model per Million mapped reads (RPKM). Based on the expression levels, the significant DEGs (Differentially expressed genes) among different samples were identified with p-value ≤0.05 and log_2_fold-change ∣log 2 FC∣ ≥1. The cluster of the DEGs was performed by using the common perl and R scripts.

### Functional enrichment analysis

DEGs were converted to human orthologous genes and submitted to DAVID web server (http://david.abcc.ncifcrf.gov/) for enrichment analysis of the significant overrepresentation of GO biological processes (GO-BP), molecular function (GO-MF) terminologies, and KEGG-pathway category. In all tests, the *P*-values (i.e. EASE score) were calculated using Benjamini-corrected modified Fisher’s exact test. Only the *P*-values less than 0.05 were considered as significant and listed. Finally, specific expression transcripts were compared to gene and QTL mapping results in the AnimalQTLdb (PigQTLdb: http://www.animalgenome.org/QTLdb/pig.html) to explore their enrichment. A QTL gene was defined as an overlapping region with QTL regions, and the overlapping region was at least half the length of the gene or the QTL region (< 2 Mb).

### Quantitative RT-PCR

Quantitative RT-PCR (Q-PCR) was used to measure the mRNA expression levels of six representative genes. The Q-PCR was performed using the SYBR Green Real-time PCR Master Mix (TaKaRa, China) on a CF96 Real-Time PCR Detection System (Bio-Rad). The PCR primer sequences are shown in S9 Table. *ACTB*, *TBP* and *TOP2B* genes were simultaneously used as internal gene for normalization. The 2^-ΔΔCt^ method was used to determine the relative mRNA abundance[23].

## Results and Discussion

### Sigmoidal growth curve of Liangshan pig

**Fig 1:**
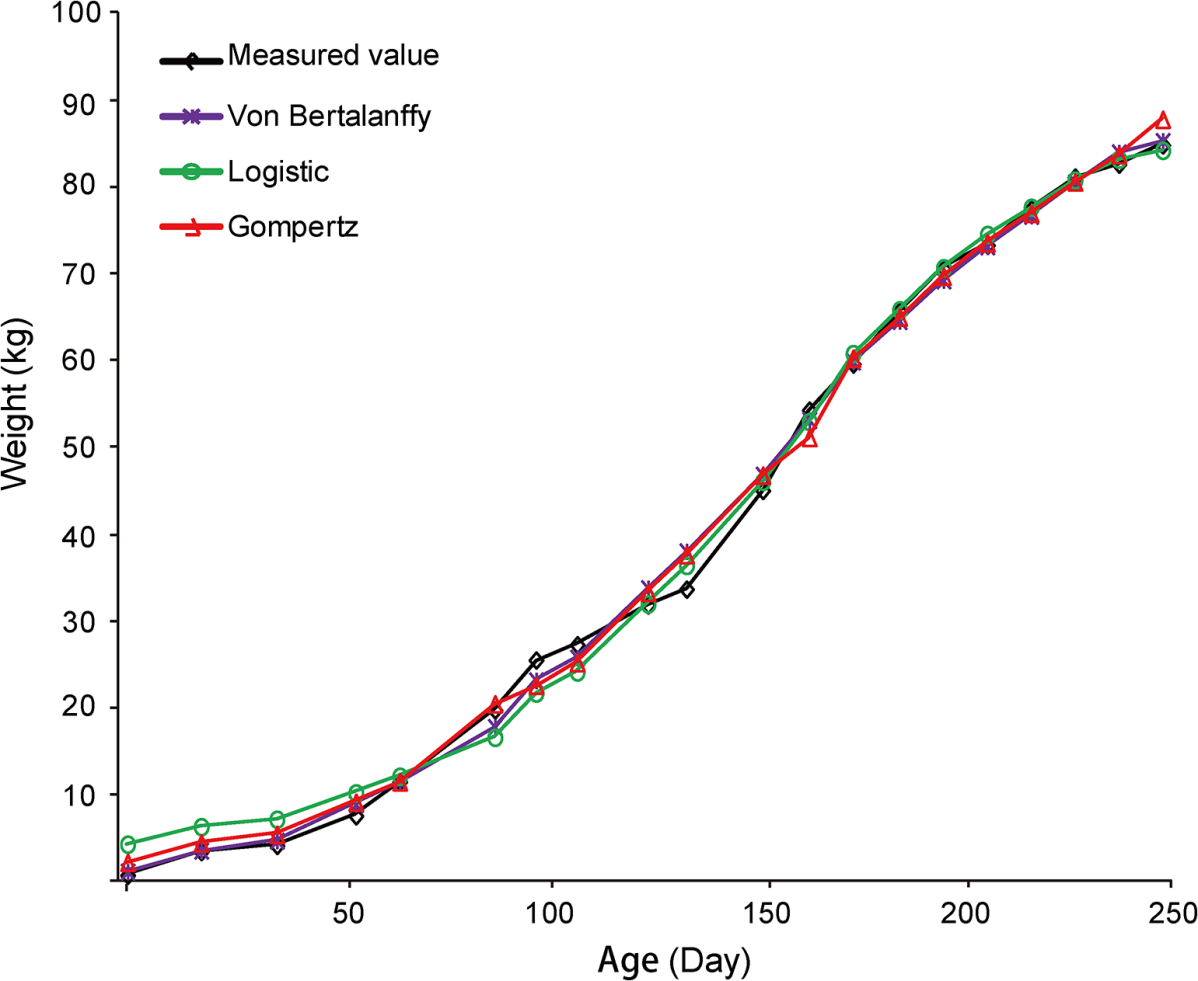
Growth curves of Liangshan pigs.

To predict the growth inflection point of Liangshan pigs for transcriptome analysis, we recorded the growth data of 275 female Liangshan pigs from birth to 250 days of age (S1 Table). Subsequently, three frequently used growth functions were fitted to the data and their accuracy of prediction was compared (Fig 1 and S2 Table). We found that all three models had a good fit with a typical sigmoidal curve. The Von Bertalanffy curve has the highest goodness of fit (R^2^ = 0.9971) (S2 Table). The inflection point analysis of the growth curve suggested that Liangshan pigs reached their maximum growth rate at day 193.40 and the average body weight at this time was 62.61 kg. Liangshan pigs reached their maximum growth rate later and at a small body weight than Durocs, who reached their maximum growth rate at 163.6 days and 134.6 kg on average [4]. The maximum growth rate of Liangshan pigs was 455.43 g/d, much lower than ‘Pietrain’ type pigs (‘Pietrain’ type progeny were 50% Pietrain, 25% Landrace, and 25% Large White), which had a maximum growth rate of 960 g/d at 68 kg live weight [24]. These results suggested that as a typical mountain-type pig breed, Liangshan pig has a lower growth rate than foreign breeds, which was related to the lack of long-term artificial selection for the purpose of growth. Based on the growth curve, we selected 9 Liangshan pigs for subsequent transcriptome analysis at the inflection point and asymptote stages. The weight and age information of these pigs was shown in S3 Table.

### Differences in phenotypic traits

To explore the development of phenotypic traits, we slaughtered Liangshan pigs in seven additional growth stages (S4 Table). Carcass traits (backfat thickness, *longissimus dorsi* muscle area, body length, dressing percentage, and fur and fat percentage) tended to increase with increasing slaughter weight. However, lean and bone percentage increased to a point and then declined with weight and had a trend similar to that of the growth curve (S1 Fig). pH_1_, pH_2_, intramuscular fat (IMF) content and marbling score increased with weight gain, but L_1_, L_2_ and drip loss decreased with weight gain. pH is a major indicator of meat quality, which affects not only technical but also eating quality of pork [21]. IMF content influences juiciness, tenderness and meat flavor. These results indicated that Liangshan pigs have superior meat quality at an older slaughter age (S5 Table).

Intramuscular fatty acids (IFA) and free amino acids (FAA) are also important meat quality characteristics. The thermal degradation of fatty acid and Maillard reactions between FAA and reducing sugars are the main sources of aromatic volatiles in cooked meat [25]. Therefore, we measured IFA and FAA composition of the *longissimus dorsi* muscle at the inflection point and asymptotic stages of the growth curve. Total fatty acid content was 1493.13 mg/100 g, 2019.11 mg/100 g and 2451.95 mg/100 g BIP, UIP and AIP, respectively. These results were in agreement with the uptrend in IMF in the three development stages. C16:0, C18:0, C18:1, and C18:2 were the main FAs (>80%). The ratio of polyunsaturated fatty acids (PUFA) to saturated fatty acids (P:S) was greater than 0.4 (S6 Table). Similar results were reported in other native Chinese pig breeds [21]. However, most of the FAA concentrations were significantly higher at UIP than BIP or AIP, which does not agree with the generally accepted idea that almost all FAA increase with slaughter age (S7 Table). However, tthis result was in accordance with a previous report [26], which found that FAA in cattle were higher at 25 months than 15 and 35 months, and were correlated with the growth rate lower at 15 or 35 months old than 25 month. Therefore, the daily gain of Liangshan pigs at AIP may be a result of the accumulation of fat, higher FAA at UIP probably caused by the higher growth rate of muscle.

Based upon the meat quality characteristics found at the three development stages, this study confirmed that Liangshan pigs have good meat quality and increasing the slaughter age could be used in production systems to provide better taste and flavor quality to meat.

### Summary and validation of DGE profiling libraries

To identify genes in response to the differing physiology of Liangshan pigs between the inflection point and asymptotic stages, RNA libraries were constructed using the *longissimus dorsi* BIP, UIP and AIP (Table 1). The DGE libraries generated a total of 117 million single-end reads of 50 bp. The total reads length was 5.89 gigabases, representing about two times the size of the pig genome. About 64.2%- 67.9% of all reads were well aligned to the UCSC pig reference genome (Sus scrofa 10.2) using the bowtie package. 51.24%- 53.76% of reads had a unique genomic location and 0.18%- 0.25% of reads were mapped to the antisense strand. Individual transcripts were calculated and normalized by FPKM (the number of fragments per kilobase of exon per million fragments mapped). If one transcript was expressed in at least one of the 9 libraries, we labeled it as an expressed transcript and used for subsequent analysis [27]. Consequently, we identified 21,331, 20,996, and 20,139 expressed transcripts BIP, UIP and AIP, respectively (Table 1).

**Table 1:** Summary of transcriptome alignment.

Group	BIP-1	BIP-2	BIP-3	UIP-1	UIP-2	UIP-3	AIP-1	AIP-2	AIP-3
Total raw reads	13,244,525	10,263,834	17,662,944	11,838,415	12,297,883	13,430,696	13,085,474	12,601,514	12,886,097
Total aligned reads	8,687,301	6,593,554	11,811,315	7,738,479	8,339,694	8,988,038	8,813,989	8,561,995	8,597,073
Match(uniqe Sense)< = 1 mismatch									
1 unique seq-> 1 gene	6,907,836	5,235,205	9,306,149	6,130,245	6,382,666	7,022,047	6,883,027	6,750,551	6,732,015
1 unique seq-> n gene	1,664,320	1,266,659	2,379,772	1,511,106	1,868,385	1,864,979	1,831,504	1,713,144	1,767,300
Match(uniqe Antiense)< = 1 mismatch									
1 unique seq-> 1 gene	30,117	23,601	27,725	22,421	22,902	26,306	21,715	24,060	23,455
1 unique seq-> n gene	2,444	2,114	2,921	2,392	1,904	2,316	1,961	2,019	2,067
Match(both Sense and Antiense)	82,584	65,975	94,748	72,315	63,837	72,390	75,782	72,221	72,236
Expressed transcripts	20,690	19,907	20,904	19,907	19,823	19,599	19,514	19,263	19,599

BIP: before inflection point; UIP: under inflection point; AIP: after inflection point.

Among the transcript populations, only a small number of genes were highly expressed (S2 Fig). The highest content transcript occupied 9.64% of the total transcripts, and the top ten highest abundance transcripts contributed approximately 30.9%. Seven of the top ten genes were coexpressed in the three development stages and had a stable expression level (S3 Fig), which may play cellular housekeeping gene roles and regulate myogenesis and basal cellular metabolism. One such example is the actin α1 (*ACTA1*), which plays a key role in forming the thin filament core and producing the force of muscle contraction [28].

**Fig 2:**
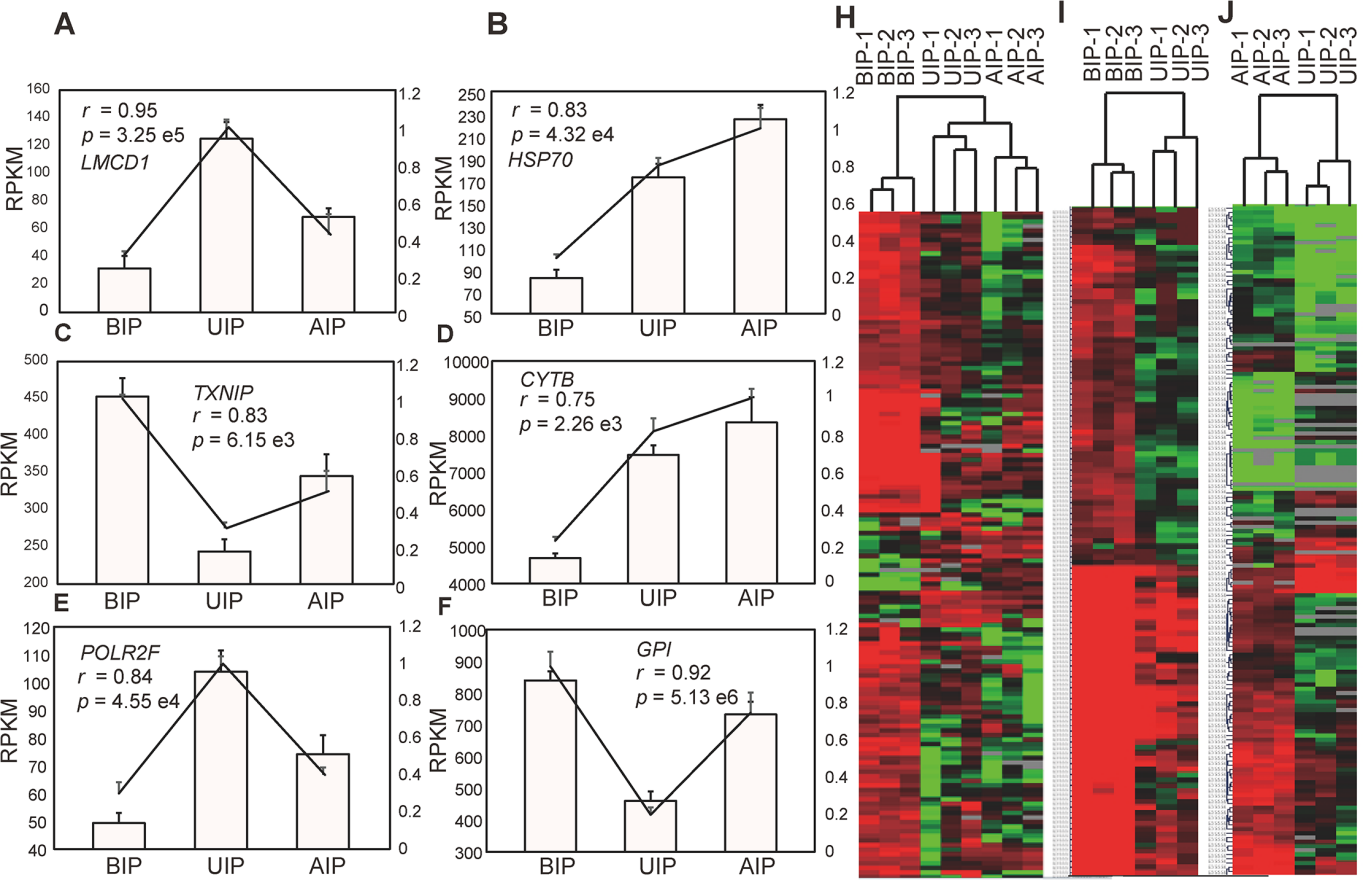
The reliability and reproducibility of DGE. (A-F) The correlation between DGE and Q-PCR. *LMCD1*: LIM and cysteine-rich domains 1, *HSP 70*: heat shock protein 70, *TXNIP*: thioredoxin interacting protein, *CYTB*: cytochrome b, *POLR2F*: polymerase (RNA) II (DNA directed) polypeptide F, *GPI*: glucose-6-phosphate isomerase. The Pearson correlation coefficient (r) and the corresponding significance value (P) were shown above the histogram. (H-J): Hierarchical clustering analysis for biological reproducibility. BIP: before inflection point, UIP: under inflection point, AIP: after inflection point.

To further validate the high throughput sequencing results, the expression patterns of 6 randomly selected transcripts in each of the three development stages were validated by Q-PCR. The results indicated that the expression patterns of these genes were highly consistent between the two methods (Pearson’s *r* > 0.75, *P* < 0.01; Fig 2A–2F). The saturation and distribution of clean reads expression were analyzed to evaluate the quality of the DGE libraries (S4–S6 Figs). The reproducibility and reliability of transcriptome libraries were analyzed by differential gene expression using hierarchical clustering. As shown in Fig 2H–2J, the three biological replicates of each stage were highly correlated with one another (Pearson’s *r* > 0.96, S7 Fig) and all the three libraries of each stage could be clearly assigned to a group. This verified the high reproducibility and reliability of DGE profiling in this study.

### Differential gene expression analysis

**Fig 3:**
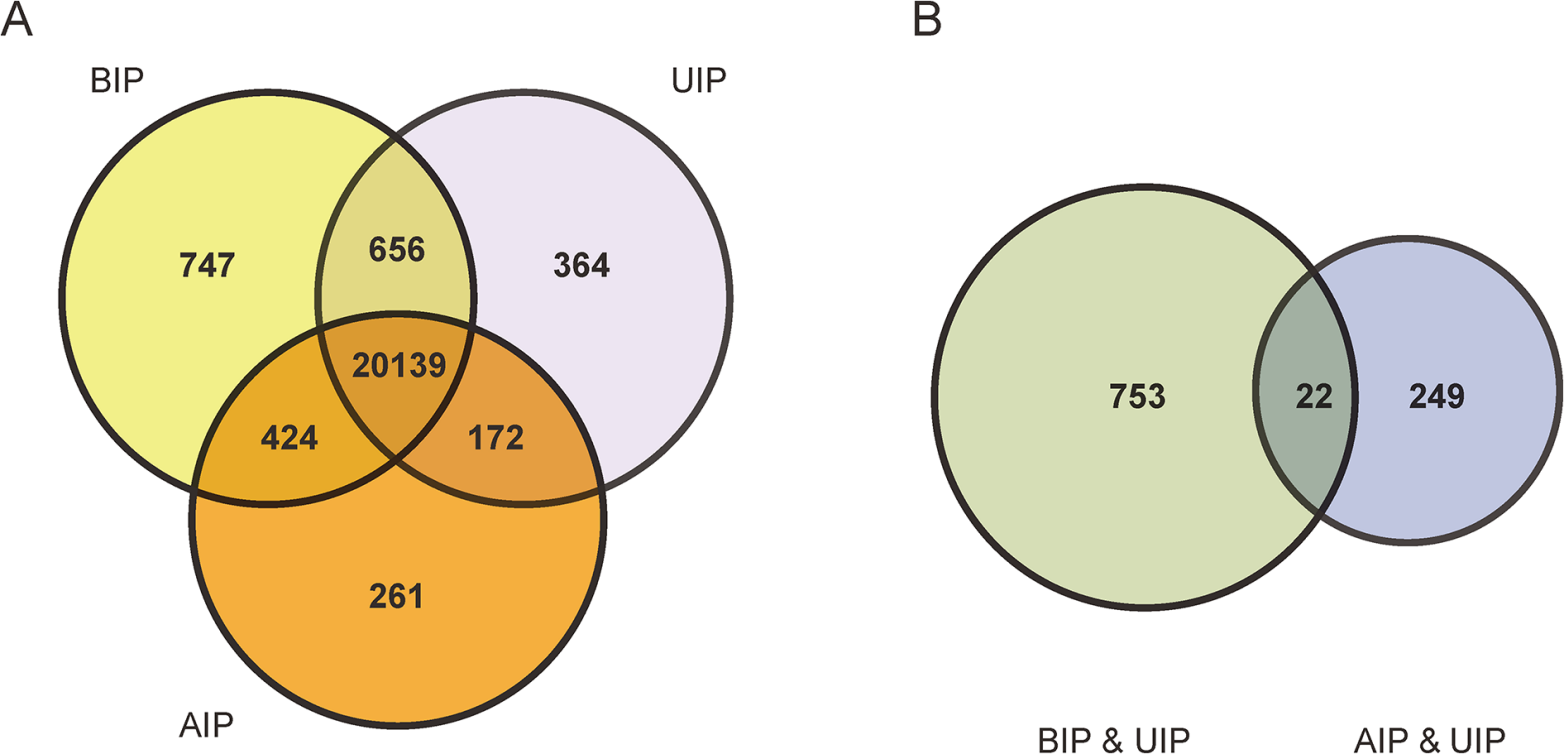
The number of expreesed genes in different development stages. (A) Distribution of expressed genes among the three development stages. (B) Distribution of different expressed genes between ‘BIP vs UIP’ and ‘AIP vs UIP’. ‘BIP vs UIP’: The number of DEGs between BIP and UIP, ‘AIP vs UIP’: The number of DEGs between AIP and UIP.

**Fig 4:**
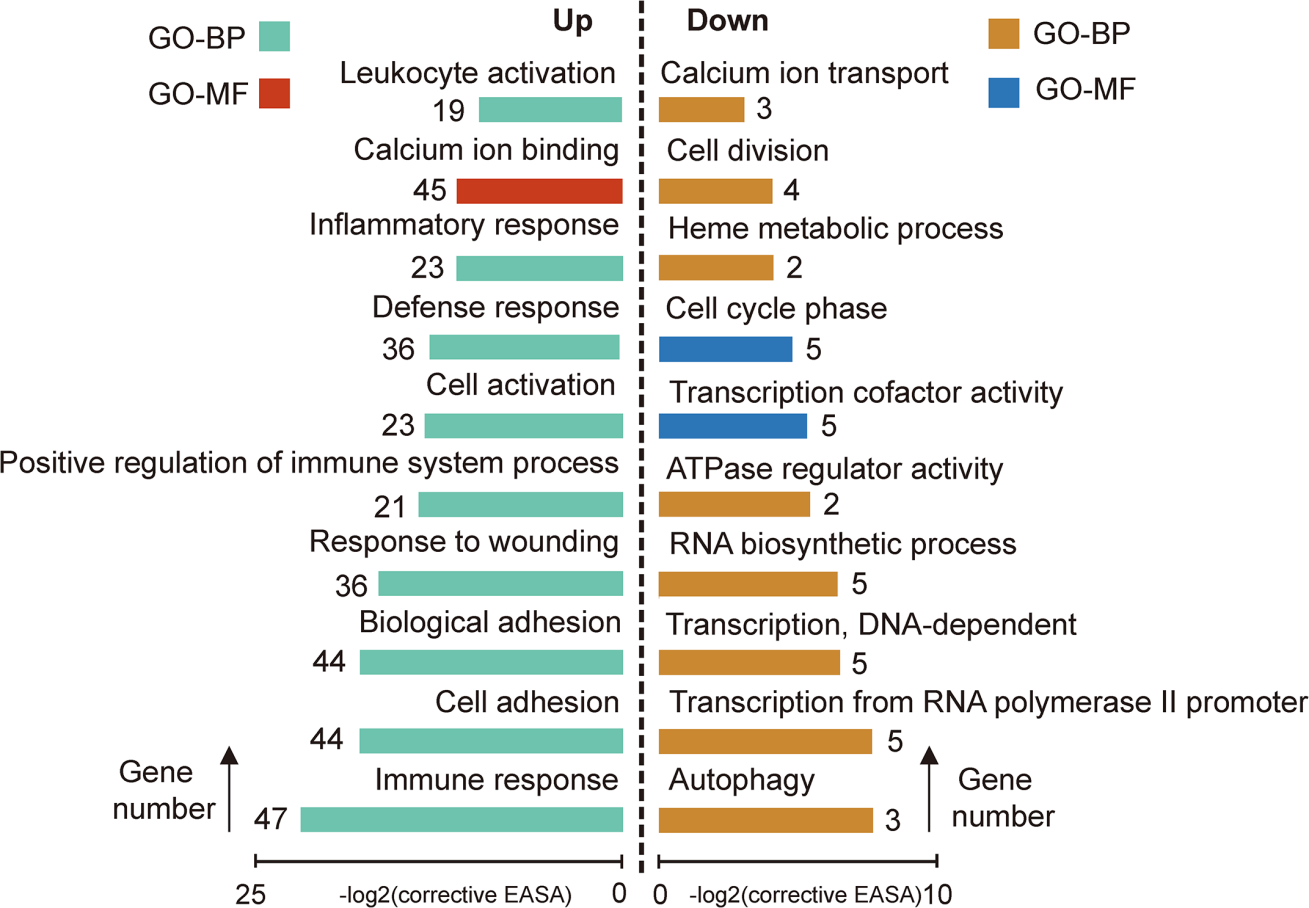
Gene Ontology (GO) categories enriched for DEGs between BIP and UIP. UP: genes higher expressed in BIP, Down: genes higher expressed in UIP. The EASE score, which indicated the significance of the comparison, was calculated by Benjamini-corrected modified Fisher’s exact test. BP, biological process; MF, molecular function.“Gene number” was the number of genes that significantly enriched in each Go term.

**Fig 5:**
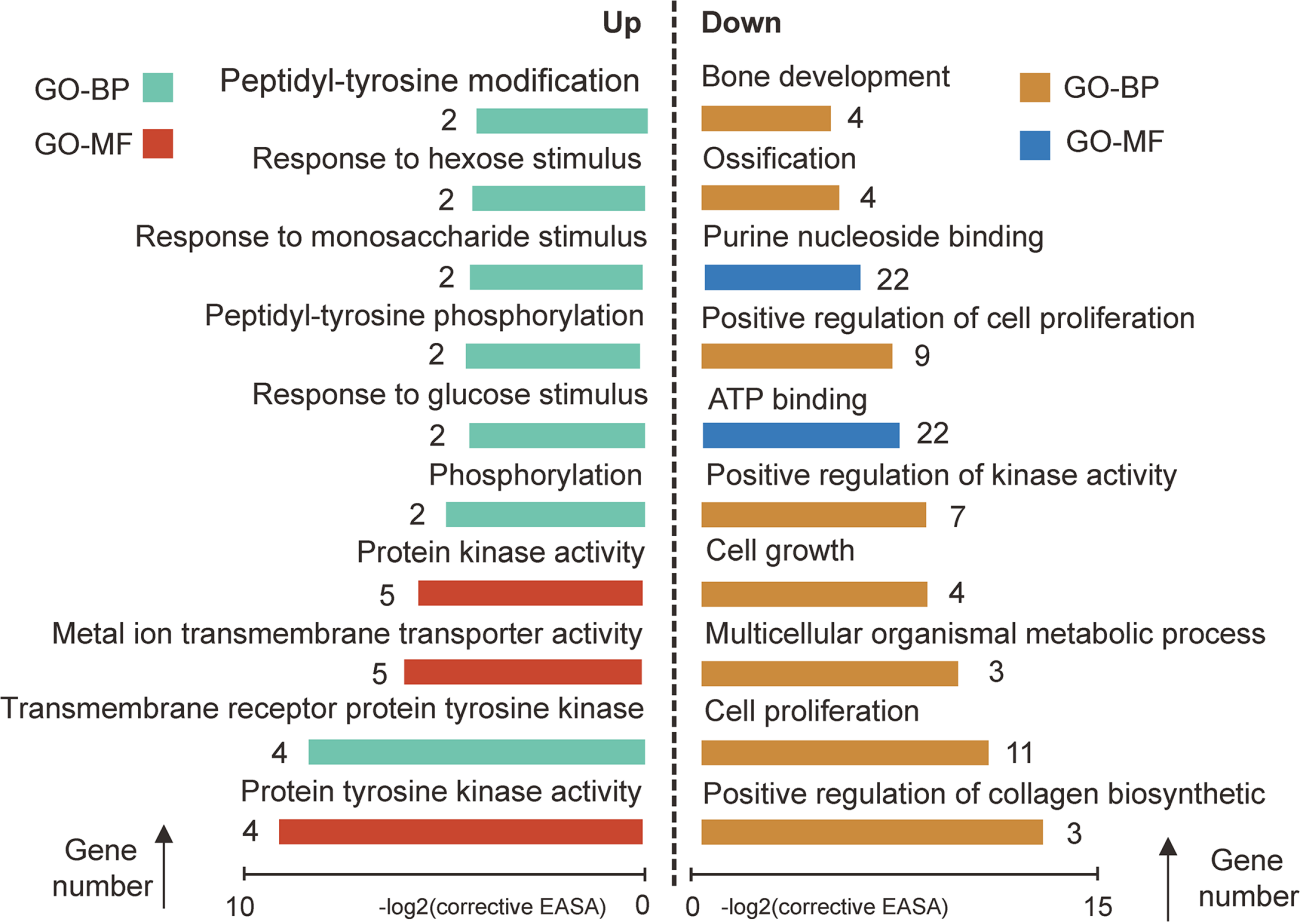
Gene Ontology (GO) categories enriched for DEGs between AIP and UIP. UP: genes higher expressed in AIP, Down: genes higher expressed in UIP. The EASE score, which indicated the significance of the comparison, was calculated by Benjamini-corrected modified Fisher’s exact test. BP, biological process; MF, molecular function. “Gene number” was the number of genes that significantly enriched in each Go term.

To explore the global transcriptional changes, we identified a total of 20,139 transcripts which were co-expressed in all three developmental stages (Fig 3A). These transcripts were primarily enriched in the categories of ‘nucleotide binding’ (1,735 transcripts, P = 6.64E-38), ‘purine nucleotide binding’ (1,482 transcripts, P = 1.82E-31), ‘adenyl nucleotide binding’ (1,233 transcripts, P = 2.27E-29) (Fig 3A). These co-expressed genes were related to basal molecular function of the cell, and results were in agreement with previous findings [29]. Furthermore, we identified 775 differentially expressed genes between BIP and UIP (Fig 3B). The higher expression genes in BIP were primarily enriched in the categories of ‘Immune response’ (47 transcripts, P = 2.36E-09), ‘response to wounding’ (36 transcripts, P = 2.83E-07), ‘positive regulation of immune system process’ (21 transcripts, P = 3.36E-06). The more highly expressed genes in UIP were primarily enriched in the categories of ‘cell cycle phase’ (5 transcripts, P = 1.29E-04), ‘transcription from RNA polymerase II promoter’ (7 transcripts, P = 5.32E-04), ‘RNA biosynthetic process’ (5 transcripts, P = 7.39E-03) (Fig 4). In addition, there were only 271 DEGs identified between AIP and UIP. The more highly expressed genes in AIP were primarily enriched in the categories of ‘Protein kinase activity’ (5 transcripts, P = 1.69E-04), ‘Phosphorylation’ (8 transcripts, P = 3.18E-04), ‘Protein tyrosine kinase activity’ (6 transcripts, P = 1.09E-05). The higher expression genes in UIP were primarily enriched in the categories of ‘positive regulation of collagen biosynthetic process’ (9 transcripts, P = 1.60E-06), ‘ATP binding’ (22 transcripts, P = 3.80E-04), ‘ossification and bone development’ (8 transcripts, P = 2.45E-04) (Fig 5). These results closely associated with the phenotypic differences in Liangshan pigs at multiple stages of development. For example, the bone ratio increased to a point, then decreased with increasing slaughter weight, with the maximum growth rate achieved around the inflection point (S1 Fig). Simultaneously, the genes with greatest UIP expression were significantly enriched in regulatory pathways for calcium ion transport, collagen biosynthesis, ossification and bone development. Calcium ions and collagen play an important role in the composition of bone [30,31]. The result was in accordance with previous findings, which reported that crossbred pigs (¾ Landrace ×¼ Large White) had the highest daily gain at 80 kg (occurring at the inflection point), and the bone percentage increased to the inflection point (80 kg) and decreased from there [32]. The increase in the bone percentage before UIP may also be related to the change in bone mineral density (BMD). Lu *et al*. found human BMD increased significantly with age until 17.5 years in males and 15.8 years in females [33].

### Differential expression of genes related to immune system development

**Fig 6:**
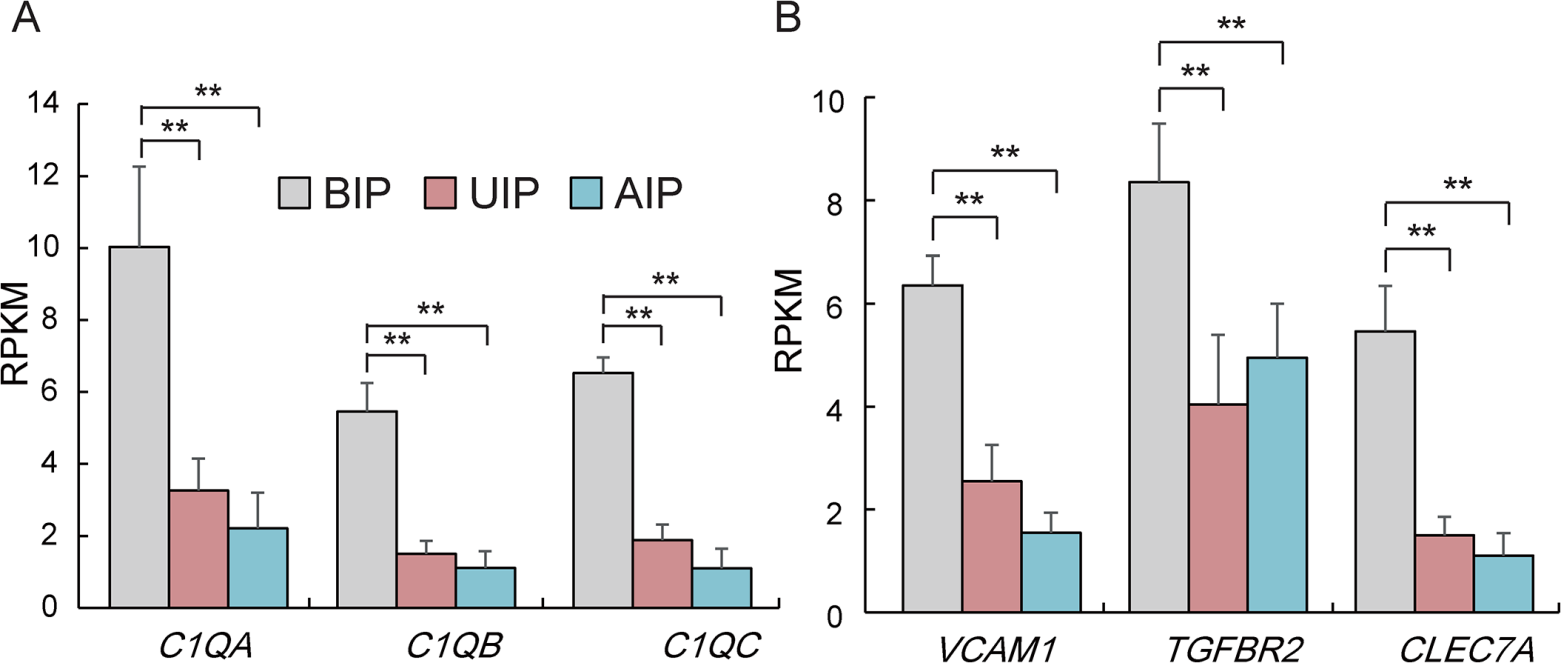
The expression level of genes related to innata immunity and immune response. (A) The expression level of innata immunity genes. (B) Gene expression level about immune response. Data are means ± SD. Statistical significance was calculated by one-way repeated-measures analysis of variance (n = 3 per individual).* p < 0.05, ** p < 0.01.

To characterize BIP immune system development in Liangshan pigs, we further detected the mRNA expression levels of six representative indicators related to innate immunity and immune response. The three subunits (*C1qa*, *C1qb* and *C1qc*) of C1q (complement component 1, q subcomponent) were more highly expressed in BIP than UIP and AIP (Fig 6A). C1q is the first subcomponent of the classical complement pathway [34], which is an important component of the innate immune system [35,36]. Moreover, the other immune response genes (*Vcam1*, *TGF*β and *Clec7a*) showed the greatest expression in BIP (Fig 6B). *Vcam1* (Vascular cell adhesion molecule-1) is a member of the immunoglobulin super-family, which acts in immune response by regulating the migration of leukocyte from the blood into the tissue infected by antigens [37–39]. The function of TGF-β in the immune system is to maintain its tolerance by regulating the proliferation, differentiation, and survival of lymphocytes [40]. Dectin-1, also known as C-type lectin domain family 7, member A (CLEC7A), which acts as a kind of pattern-recognition receptor, is able to recognize a special glycosyl sequence of exogenous antigen. In this way, *Dectin-1* plays an important role in innate immune responses [41]. Interestingly, Sonck *et al*. reported porcine *Dectin-1* only to be expressed in the small intestine, spleen, and lungs, but not in the liver, kidneys or heart [42]. In our study, we detected enrichment of the *Dectin-1* expression profile in porcine muscle. All of these results indicated that the immune system of Liangshan pigs remained in highly active status in BIP (the early developmental stage). The result was in accordance with a previous report, which found that the immune system of Zebrafish was morphologically and functionally mature by 4–6 weeks post-fertilization (wpf) [43]. 4–6 wpf represents the juvenile stage in Zebrafish, which correlates with the BIP stage in Liangshan pigs.

### Differential expression of genes related to energy metabolism, transcription and translation activity

**Fig 7:**
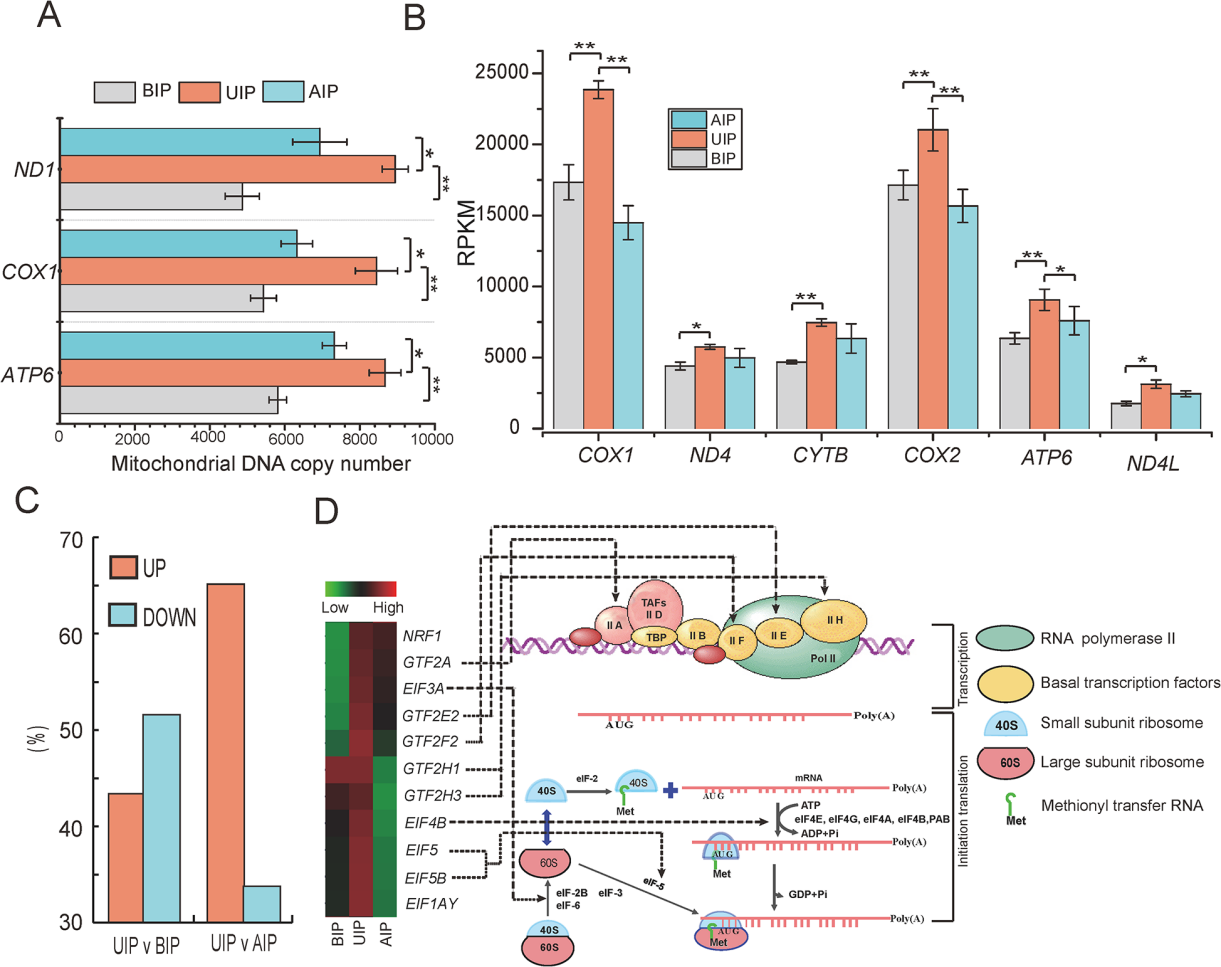
Analysis of genes involved in transcriptional activity among the three development stages. (A) The relative mitochondrial DNA copy number. (B) The mitochondrial genes expression. (C) The proportion of change of global gene expression. ‘UIP v BIP’ means the all DEGs between UIP and BIP, ‘UP’ is the proportion of high expressed in UIP of all DEGs. (D) The expression of general initiation transcription factors and translation initiation factor.

Liangshan pigs reached their maximum growth rate and daily gain at the inflection point of the growth curve. This implies that the rate of metabolism for biosynthesis is highest at UIP in Liangshan pigs. Mitochondria are the source of energy for metabolism, and play a key role in the regulation of metabolism, cell-cycle, and muscle development [44]. We utilized three mitochondrial DNA genes (*ND1*, *COX1* and *ATP6*) to measure the mtDNA copy number by Q-PCR. All the results showed a similar trend, and the mtDNA copy number was higher in UIP than AIP and BIP (Fig 7A). In addition, six randomly selected mitochondrial genes (*COX1*, *ND4*, *CYTB*, *COX2*, *ATP6* and *ND4L*) also showed greater expression in UIP than AIP and BIP (Fig 7B). These results suggested that Liangshan pigs have the highest energy cost at the inflection point.

Eukaryotic gene expression has an enormous energy cost, which consumes about75% of the ATP cellular energy budget for mRNA and protein polymerization [45]. This suggested that Liangshan pigs have global transcriptional and translational activity at the growth curve inflection. Initiation of transcription is regulated through the formation of a complex polymerid containing RNA polymerase II and the general transcription factors (GTFs), which act on the DNA promotor [46]. The process of initiating translation involves the formation of a ribosomal initiation complex which is regulated by numerous eukaryotic initiation factors (EIFs) [47]. Here, we detected the expression of some general initiation transcription factors (*GTF2A*, *GTF2E*, *GTF2F*, *GTF2H*) [46,48] and initiation translation factors (*EIF3*, *EIF4*, *EIF5*) [47] in the three developmental stages. The expression of these genes was significantly higher in UIP (*p* < 0.05) (Fig 7D). Moreover, the other specific transcription factor (*NRF1)* also showed greater expression in UIP than BIP and AIP, which governs mitochondrial biogenesis by activating the promotor of mitochondrial transcription specificity factors (TFB1M and TFB2M) [49]. This result showed the consistency of the expression model between the *NRF1* gene and mitochondrial genes in this study. Finally, we analyzed the global gene expression variability and found that about 65% of genes had higher expression in UIP than AIP and 43% of genes had higher expression in UIP than BIP (Fig 7C). According to these results, this study confirmed that Liangshan pigs reach their maximum growth rate and daily gain at the inflection point, which contributed to the high mitochondrial copy number and elevated global transcriptional and translational activity. We also found that mitochondrial copy number was highly related to global transcriptional activity. These results were in accordance with one previous study [45], which reported that mitochondrial content can modulate the global variability in gene expression.

### Differential expression of genes related to intramuscular fat content and fatty acid composition

**Fig 8:**
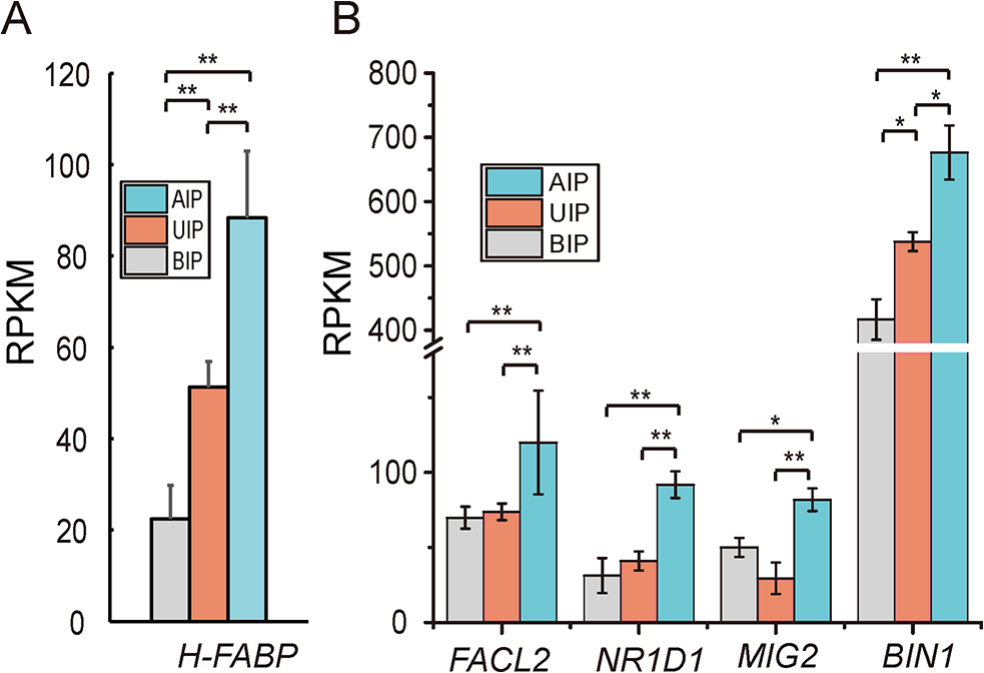
Analysis of genes involved in intramuscular fat and fatty acid synthesis. (A) Intramuscular fat synthesis related genes expression. (B) Fatty acid synthesis related genes expression. Data are means ± SD. Statistical significance was calculated by one-way repeated-measures analysis of variance (n = 3 per individual).* *p* < 0.05, ** *p* < 0.01.

IMF content is an important meat quality trait, which correlates with meat tenderness, juiciness, and taste [50]. In this study, IMF content was increased with increasing body weight in Liangshan pigs (S5 Table). IMF content was also correlated with expression of fat synthesis related genes, such as heart type fatty acid-binding protein gene (*H-PABP*) (Fig 8A). Gerbens *et al*. found that the expression levels of *H-FABP* mRNA but not protein were significantly related to IMF content [51]. However, the other fat synthesis related genes (*HSL*, *FAS*, *LEPR*) had no significant differences among the three development stages in Liangshan pigs [52,53]. This result suggested that *H-FABP* played a key role in IMF synthesis of Liangshan pigs, and increasing the slaughter weight in some extent contributed to improved meat quality.

Fatty acid composition is not only closely related with the flavor during cooking, but also play a key role in consumer’s healthy. For example, over- consumption of saturated fatty acids (SFAs) as compared to monounsaturated fatty acids (MUFAs) or polyunsaturated fatty acids (PUFAs) can increase the risk of cardiovascular disease [54]. Therefore, the recommended ratio of PUFAs to SFAs (P:S) should be more than 0.4 [50,55]. In our study, we found the ratio of P:S in Liangshan pigs was higher than 0.67 at BIP, and continued rising to 0.72 and 0.75 at UIP and AIP, respectively (S6 Table). This ratio was higher than values reported for other breeds such as the Dawu (0.50) and Dahe (0.27) [21]. We measured genes related to fatty acid synthesis (*FACL2*, *NR1D1*, *MIG12* and *BIN1*) in Liangshan pigs [56–59], and found that all genes had the highest expression in AIP. Expression trends were similar to those in total fatty acid content at different developmental stages (Fig 8B and S6 Table). In addition, the ratio of P:S was increased with weight gain, which implied that MUFA synthesis increased. *SCD1* is a key enzyme in converting SFA to MUFA, and is involved in the biosynthesis of C18:1 and C16:1. There are other important fatty acid desaturases, such as *SCD2*, *FASD1* and *FASD2* [29,60]. Wang *et al* found those genes involved in fatty acid conversion were highly expressed in adipose tissue and catalyzed the conversion of SFA to MUFA [29]. However, we found those genes had little to no expression in the muscle tissue of Liangshan pigs. This finding suggests that the biological process of converting SFA to MUFA in muscle tissue may involve some other noncanonical pathways.

### QTL enrichment analysis of specific gene expression in different development stages

To explore the potential novel transcripts related to pig growth and meat quality traits, we examined an enrichment analysis of these specific expression genes (1372) though BLAST analysis with a high confidence and narrowed (< 2 Mb) QTL interval. We found 380 (27.7%) specific expression genes had significant enrichment in QTL regions (Table 2). In the gene group specifically expressed in UIP, most genes were significantly enriched in the growth trait QTL regions. For example, *CNDP1* was localized to chromosome 1 within the interval of QTLs for daily feed intake and average daily gain. C17H20ORF86 (ENSSSCT00000034367) was localized to the interval of QTLs for body weight, age at slaughter and average daily gain. In addition, most of the genes expressed in AIP were significantly enriched in the meat quality trait QTL regions. One such gene is the FAM132A (ENSSSCT00000003700), which was localized to chromosome 6 within the interval of QTLs for drip loss, IMF content, MUFA content and P:S ratio (S8 Table). These results were correlated with the phenotypic characteristics of Liangshan pigs during each of the development stages. Carcass traits were preferentially developed during the UIP stage and meat quality traits were preferentially developed during the AIP stage.

**Table 2:** Specific expression genes distribution in chromosome and QTLs region.

Chromosome/Mitochondrion	Gene number	Specific expression genes number in QTL region	QTL region length (Mb)
1	2209	40	22.17
2	2147	46	21.48
3	1444	21	11.58
4	1254	40	20.79
5	1172	21	7.66
6	1941	30	12.68
7	1651	44	12.46
8	847	14	8.66
9	1427	14	5.30
10	524	4	2.02
11	422	2	1.68
12	1145	15	3.09
13	1534	23	14.10
14	1370	25	13.70
15	960	16	5.93
16	444	11	4.07
17	688	9	2.45
18	512	4	2.29
X	1120	1	1.70
MT	13	0	0

The statistical significance was calculated by the *χ* ^2^-test (**: *P* < 10^−4^). MT: mitochondria.

## Conclusion

In summary, we conducted a genome-wide diversity analysis between pig transcriptomes derived from *longissimus dorsi* muscle before, at, and after the growth curve inflection point. We identified various differentially expressed genes, which were potentially associated with immune system development, energy metabolism, bone development, transcriptional activity and fatty acid metabolism. We found that Liangshan pigs reached their maximum growth rate and daily gain at the inflection point, which was related to the high energy metabolism rate of global transcriptional activity. We also identified many DE genes that were significantly enriched in the QTL regions of growth and meat quality traits. This study will contribute to animal breeding and feeding strategies.

## Supporting Information

S1 Fig Carcass traits of Liangshan pigs in different development stages. (A) Backfat thinkness of Liangshan pigs in different development stages. (B) Longissimus dorsi area of Liangshan pigs in different development stages. (C) Body length of Liangshan pigs in different development stages. (D) Dressing percentage of Liangshan pigs in different development stages. (E) Lean percentage of Liangshan pigs in different development stages. (F) Bone percentage of Liangshan pigs in different development stages. (G) Fur and fat percentage of Liangshan pigs in different development stages.
(TIFF)

S2 Fig The distribution of expressed genes. (A) The cumulative percentage of all expressed genes. (B) The composition of the highest top ten expressed genes. BIP: before inflection point, UIP: under inflection point, AIP: after inflection point. *ACTA1*: actin alpha 1, *MYH1*: myosin heavy chain 1, *CKM*: creatine kinase, *PYGM*: muscle glycogen phosphorylase, *COX1*: cytochrome oxidase subunit 1, *ATP2A*: ATPase2, *MYH2*: myosin heavy chain 2.
(TIFF)

S3 Fig The expression of top seven co-expression genes. RPKM: reads per kilobase of exon model per million mapped reads.
(TIFF)

S4 Fig Saturation analysis of DGE libraries. Saturation analysis of the capacity of libraries demonstrated that newly emerging distinct reads were gradually reduced with increase in total sequence reads when the number of sequencing reads was large enough. When the number of sequencing reads reached five million, library capacity a pproached saturation. L1, L2 and L3 were the sample from BIP; L4, L5 and L6 were the sample from UIP, L7, L8 and L9 were tha sample from AIP.
(TIFF)

S5 Fig Sequencing random distribution. (TIFF)

S6 Fig Distribution of total clean reads in each library. L1, L2 and L3were the sample from BIP; L4, L5 and L6 were the sample from UIP, L7, L8 and L9 were tha sample from AIP.
(TIFF)

S7 Fig Correlation of mRNA expression among three biological replicates within nine libraries. A scatter plot and Pearson’s correlation revealed a correlation between the log10 of mRNA expression of each biological replicate. L1, L2 and L3 were the sample from BIP; L4, L5 and L6 were the sample from UIP, L7, L8 and L9 were tha sample from AIP.
(TIF)

S1 Table The slaughter weight and average daily gain (ADG) of Liangshan pigs. (DOCX)

S2 Table The parameters of three growth curve models. A is the maximum size. k is the inherent relative growth rate at the start. B is the growth curve line constant. R2 is degree of fitting. IPD: Inflection point day; IPW: Inflection point weight; IPG: Inflection point daily gain.
(DOCX)

S3 Table The information of transcriptome sequencing sample of Liangshan pigs. (DOCX)

S4 Table Information of Liangshan pigs been slaughtered. Phases 1, 4 and 9 were also used for transcriptome analysis. Phases 2, 3, 5, 6, 7, 8 and 10 were used for carcass traits and meat quality traits measured.
(DOCX)

S5 Table The meat quality traits of Liangshan pig in different stages. pH_1_ and L_1_ measured at 45 min postmortem; pH_2_ and L_2_ measured at 24 h postmortem. S.e. standard error. NS, no significant difference, *P* > 0.05; * significant at the 5% level; **significant at the 1% level; ***significant at the 0.1% level.
(DOCX)

S6 Table The fatty acid composition of Liangshan pig’s *longissimus dorsi* in different development stages. Data was shown as the percentage of fatty acid to fresh weight. SFA = saturated fatty acid; UFA = unsaturated fatty acid; TFAC = total unsaturated fatty acid. S.e. standard error. NS, no significant difference, *P* > 0.05; * significant at the 5% level; **significant at the 1% level; ***significant at the 0.1% level.
(DOCX)

S7 Table The amino acid composition of Liangshan pig’s *longissimus dorsi* in different development stages. Data was shown as the percentage of amino acid to fresh weight and LD at 10th rib. EAA = essential amino acid; TAA = total amino acid; EAA/TAA = the ratio of EAA to TAA. a,b,c Means within a row with different superscripts indicate significant differences; S.e. standard error. NS, no significant difference, *P* > 0.05; * significant at the 5% level; **significant at the 1% level; ***significant at the 0.1% level.
(DOCX)

S8 Table Specific expressed gene in different development stages overlapped QTL regions. (XLSX)

S9 Table Primer sequences used for Q-PCR. *: *ACTB* (β actin), *TBP* (TATA box binding protein), *TOP2B* (topoisomerase II β) and *GCG* (glucagon) are the endogenous control genes. $: Primer sequences used for DNA copy number Q-PCR.
(DOCX)
