# Supplementary figures and images for: Transcriptome Analysis of Liangshan Pig Muscle Development at the Growth Curve Inflection Point and Asymptotic Stages Using Digital Gene Expression Profiling
Source: PLoS One. 2015 Aug 20;10(8):e0135978. doi: 10.1371/journal.pone.0135978 (PMC4546367; doi:10.1371/journal.pone.0135978)

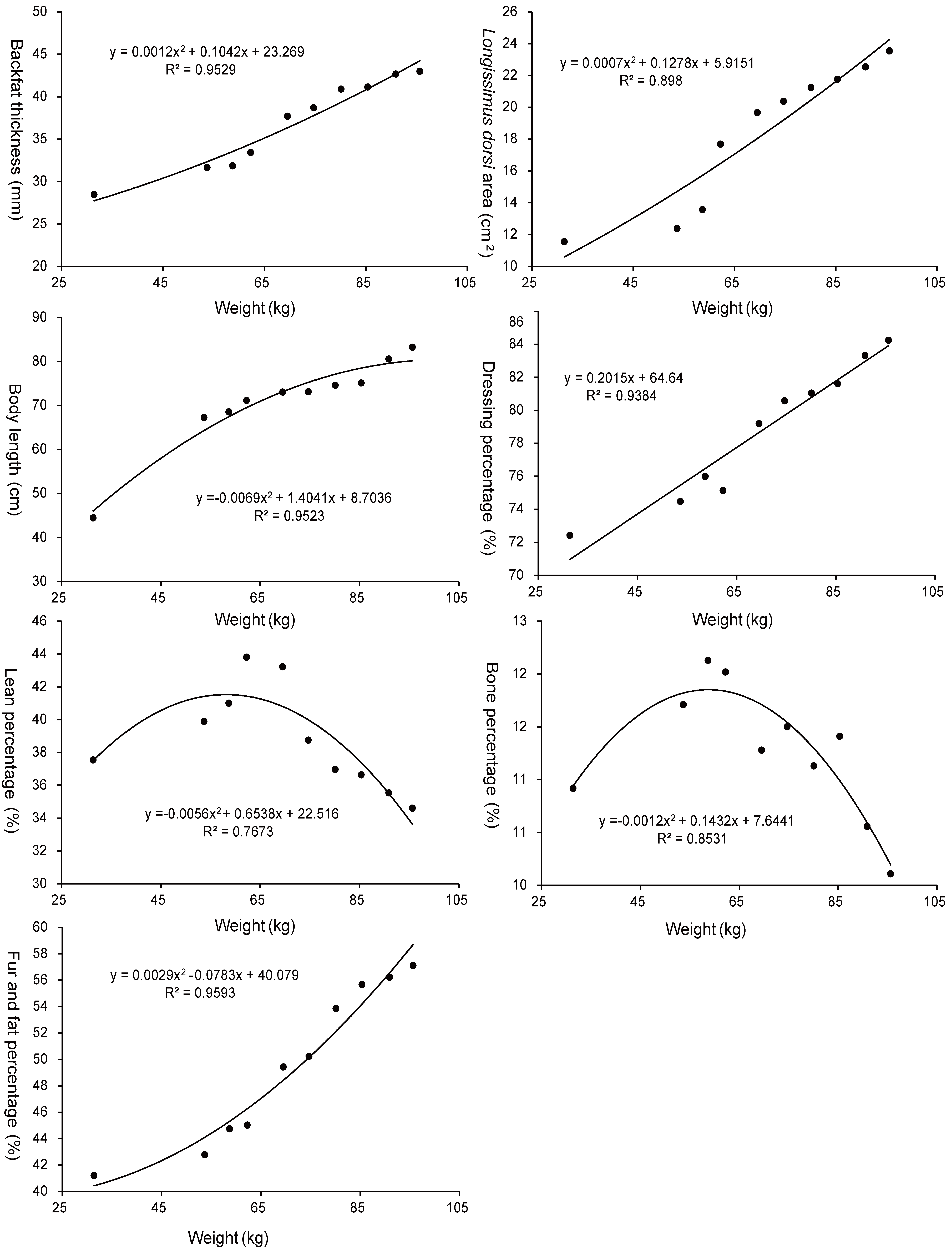

Supplement: S1 Fig — (A) Backfat thinkness of Liangshan pigs in different development stages. (B) Longissimus dorsi area of Liangshan pigs in different development stages. (C) Body length of Liangshan pigs in different development stages. (D) Dressing percentage of Liangshan pigs in different development stages. (E) Lean percentage of Liangshan pigs in different development stages. (F) Bone percentage of Liangshan pigs in different development stages. (G) Fur and fat percentage of Liangshan pigs in different development stages. (TIFF) [file pone.0135978.s001.tiff]

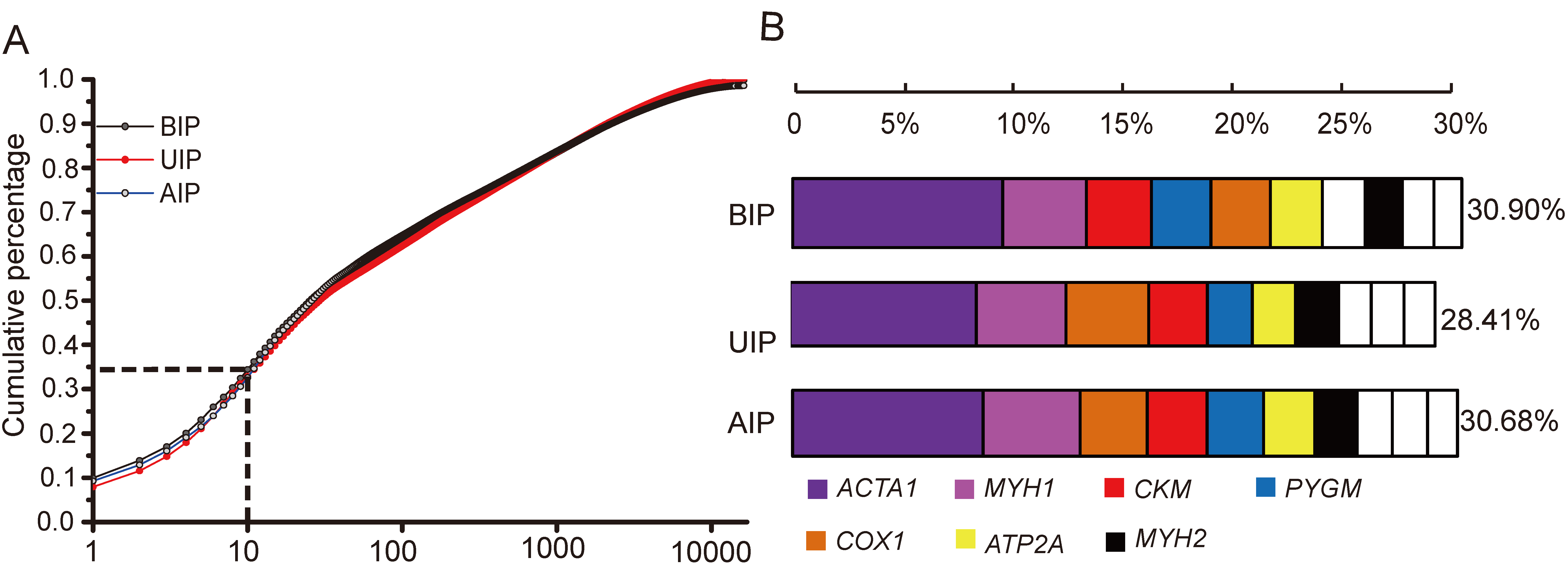

Supplement: S2 Fig — (A) The cumulative percentage of all expressed genes. (B) The composition of the highest top ten expressed genes. BIP: before inflection point, UIP: under inflection point, AIP: after inflection point. ACTA1: actin alpha 1, MYH1: myosin heavy chain 1, CKM: creatine kinase, PYGM: muscle glycogen phosphorylase, COX1: cytochrome oxidase subunit 1, ATP2A: ATPase2, MYH2: myosin heavy chain 2. (TIFF) [file pone.0135978.s002.tiff]

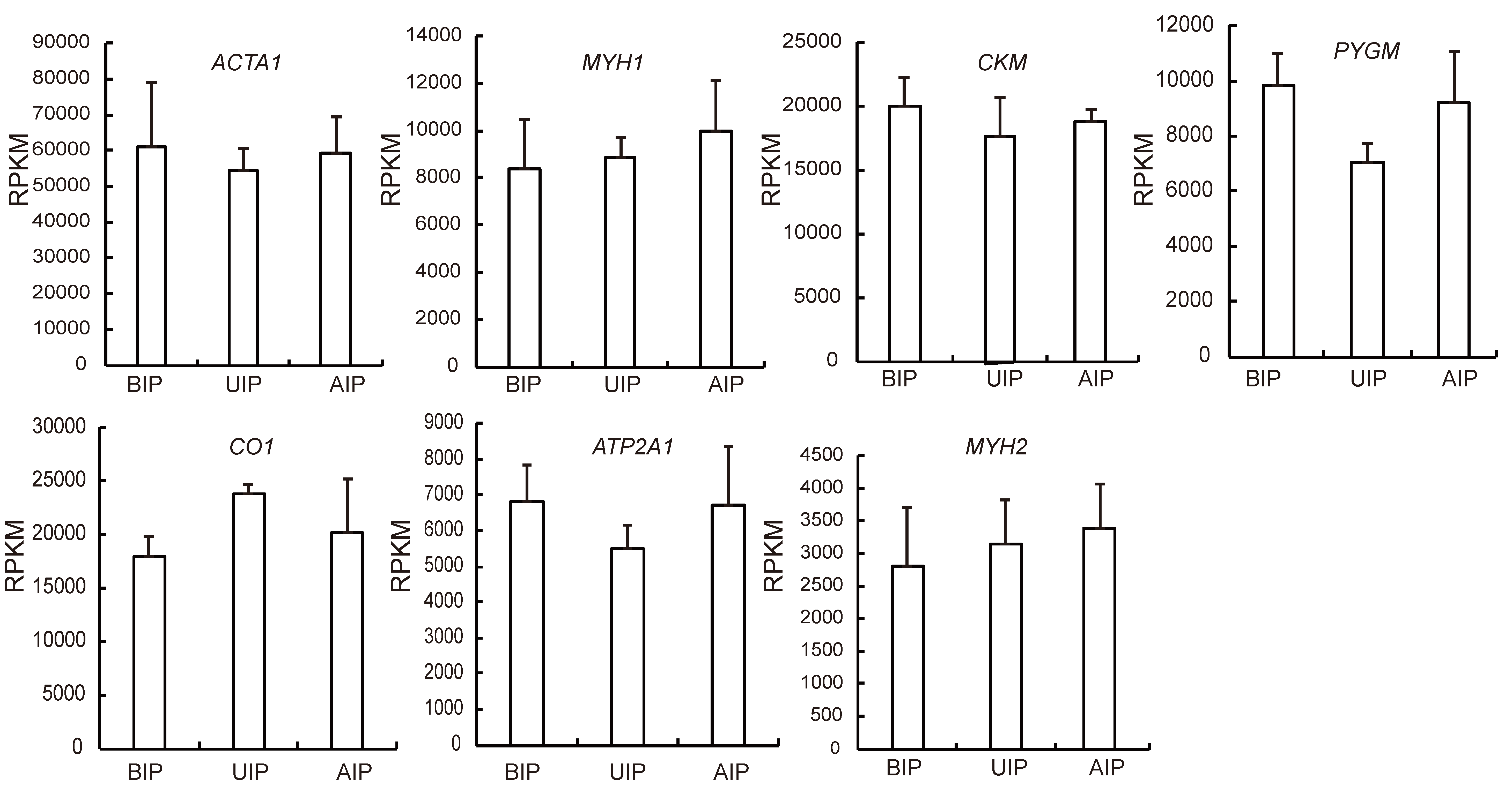

Supplement: S3 Fig — RPKM: reads per kilobase of exon model per million mapped reads. (TIFF) [file pone.0135978.s003.tiff]

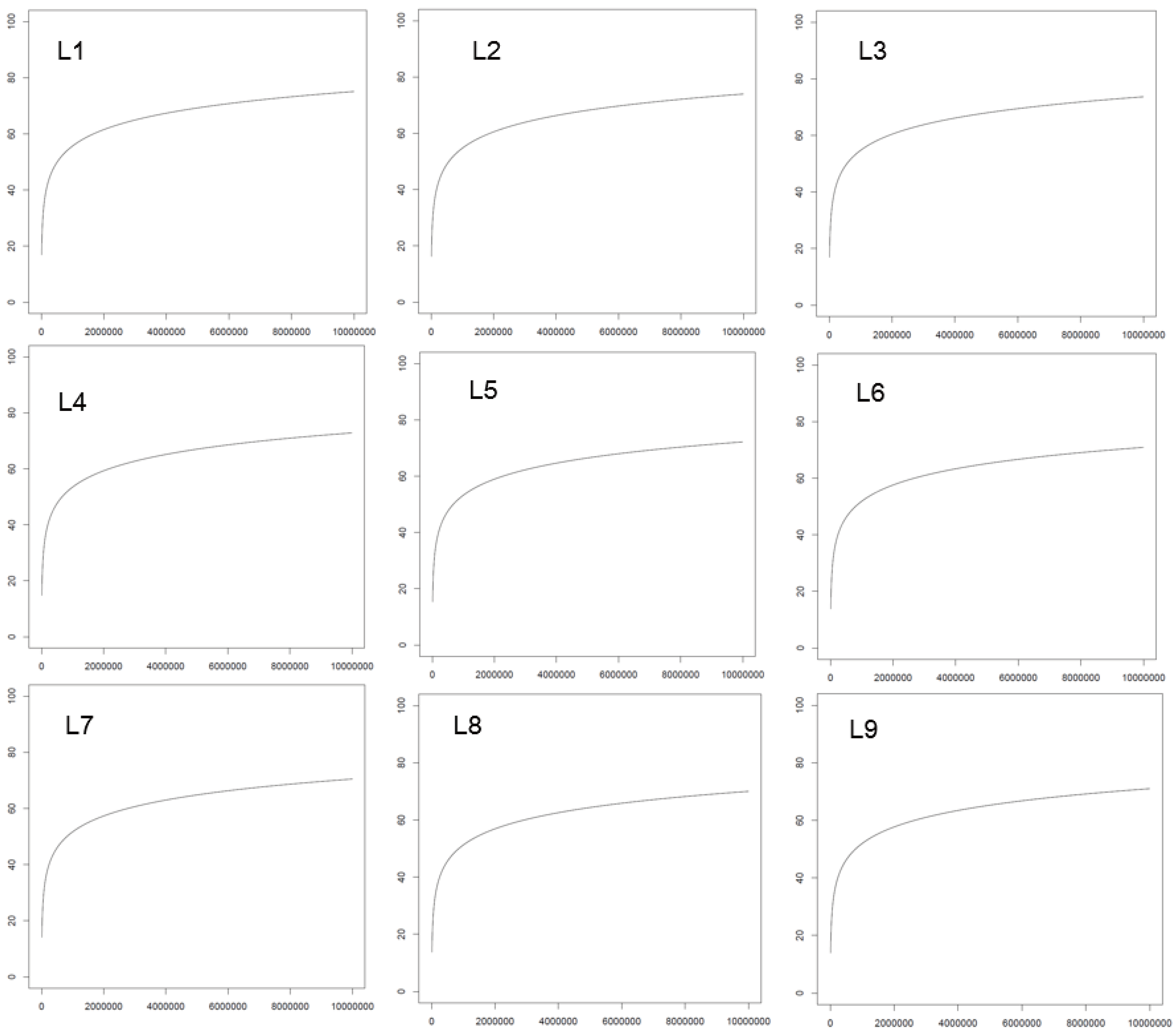

Supplement: S4 Fig — Saturation analysis of the capacity of libraries demonstrated that newly emerging distinct reads were gradually reduced with increase in total sequence reads when the number of sequencing reads was large enough. When the number of sequencing reads reached five million, library capacity a pproached saturation. L1, L2 and L3 were the sample from BIP; L4, L5 and L6 were the sample from UIP, L7, L8 and L9 were tha sample from AIP. (TIFF) [file pone.0135978.s004.tiff]

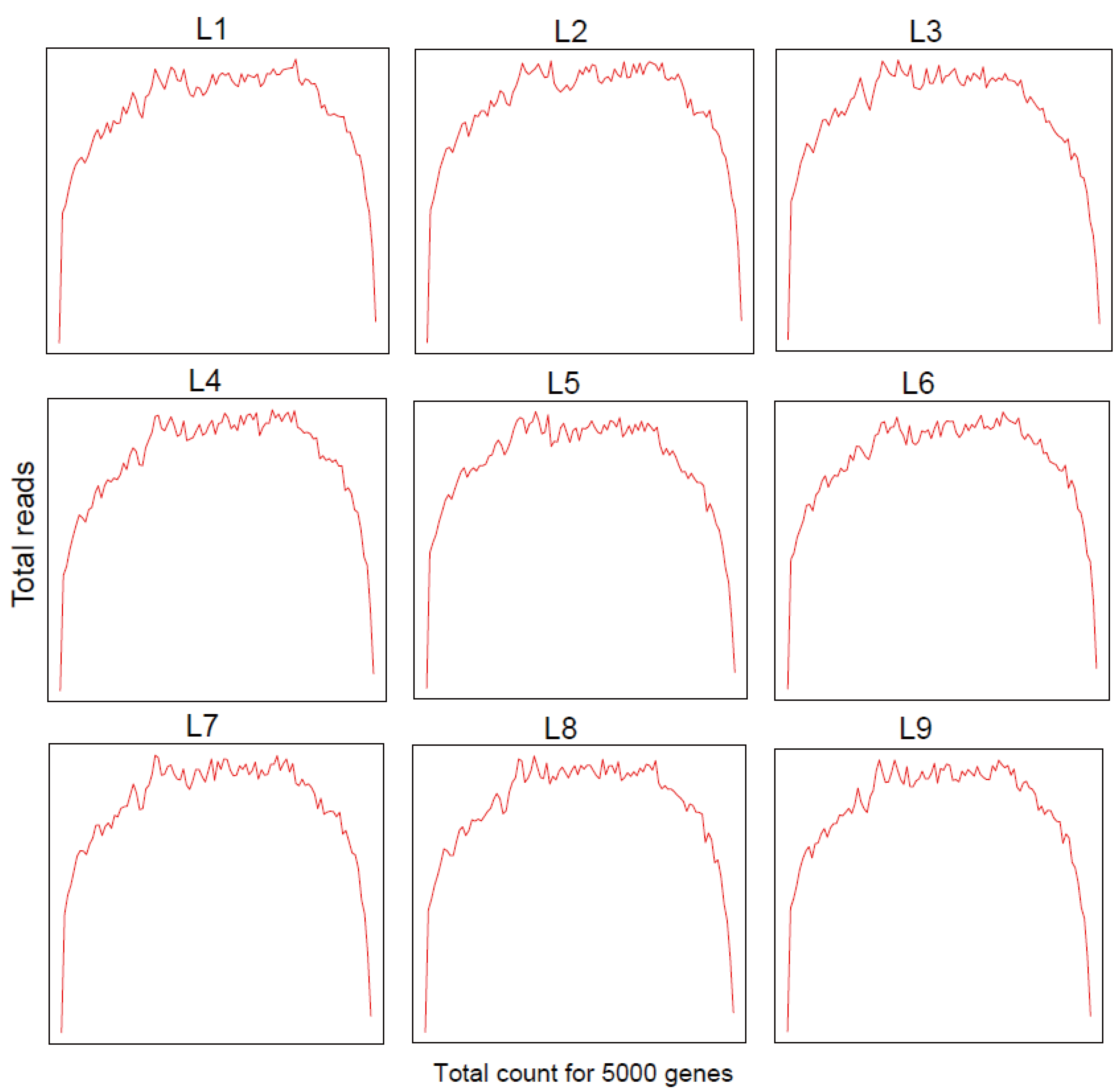

Supplement: S5 Fig — (TIFF) [file pone.0135978.s005.tiff]

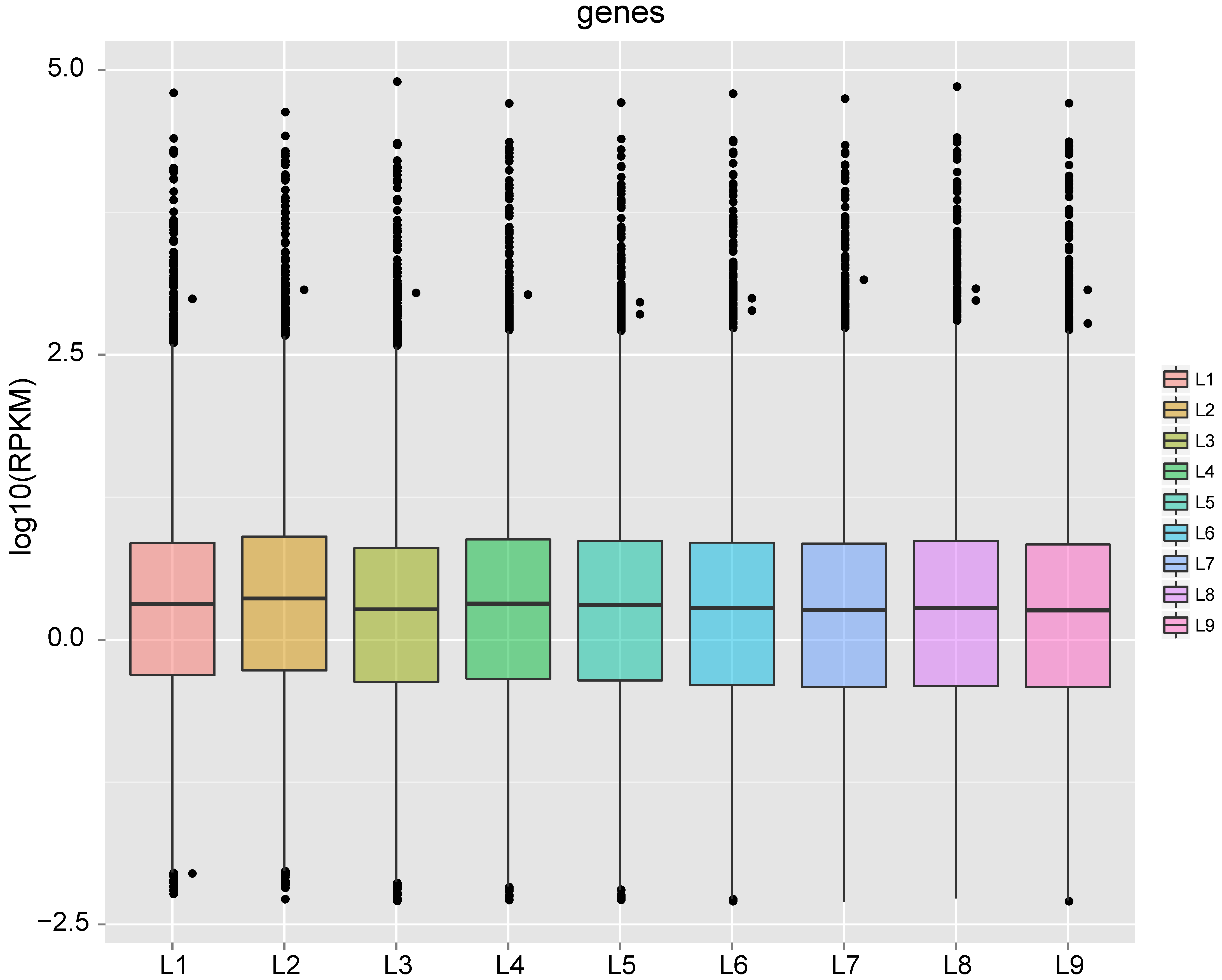

Supplement: S6 Fig — L1, L2 and L3were the sample from BIP; L4, L5 and L6 were the sample from UIP, L7, L8 and L9 were tha sample from AIP. (TIFF) [file pone.0135978.s006.tiff]

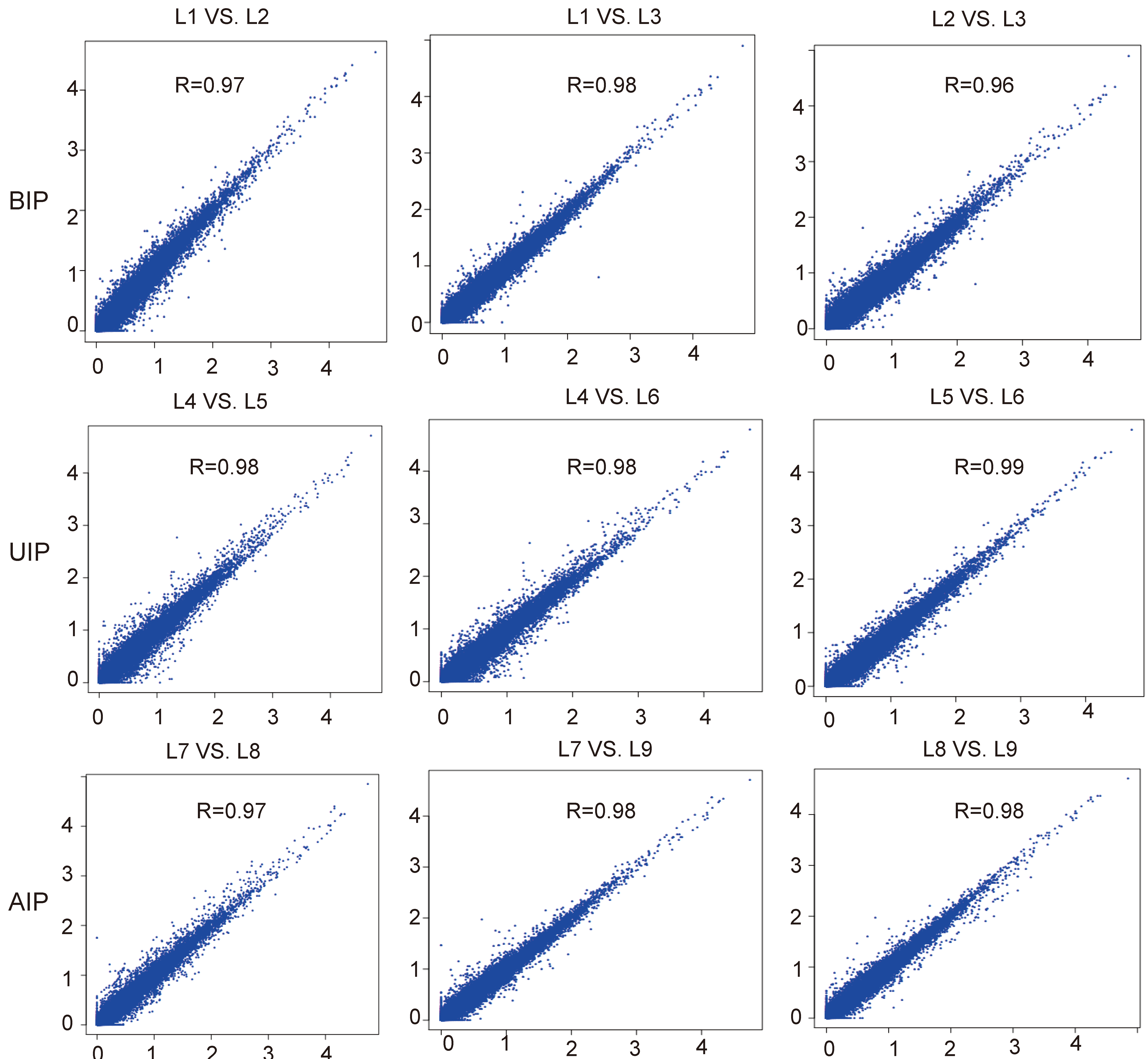

Supplement: S7 Fig — A scatter plot and Pearson’s correlation revealed a correlation between the log10 of mRNA expression of each biological replicate. L1, L2 and L3 were the sample from BIP; L4, L5 and L6 were the sample from UIP, L7, L8 and L9 were tha sample from AIP. (TIF) [file pone.0135978.s007.tif]
