# Supplementary material for: Transcriptome Analysis of Liangshan Pig Muscle Development at the Growth Curve Inflection Point and Asymptotic Stages Using Digital Gene Expression Profiling
Source: PLoS One. 2015 Aug 20;10(8):e0135978. doi: 10.1371/journal.pone.0135978 (PMC4546367; doi:10.1371/journal.pone.0135978)
Supplement: S1 Table — (DOCX) [file pone.0135978.s008.docx]

**Table S1 The** **slaughter weight and average daily gain (ADG) of Liangsh**an pigs

| **Day** | **Measured value** | | **Von Bertalanffy** | | **Logistic** | | **Gompertz** | |
| --- | --- | --- | --- | --- | --- | --- | --- | --- |
|  | **Weight (kg)** | **ADG (g)** | **Weight (kg)** | **ADG (g)** | **Weight (kg)** | **ADG (g)** | **Weight (kg)** | **ADG (g)** |
| 0 | 0.77 |  | 1.16 |  | 4.13 |  | 2.17 |  |
| 21 | 3.33 | 121.90 | 3.44 | 108.65 | 6.03 | 90.69 | 4.26 | 99.76 |
| 28 | 4.00 | 95.71 | 4.51 | 153.75 | 6.83 | 114.39 | 5.21 | 135.12 |
| 48 | 7.20 | 160.00 | 8.46 | 197.22 | 9.69 | 142.98 | 8.69 | 174.21 |
| 58 | 10.91 | 371.00 | 10.89 | 243.53 | 11.49 | 180.07 | 10.89 | 220.22 |
| 88 | 22.68 | 392.33 | 19.84 | 298.06 | 18.71 | 240.64 | 19.35 | 282.04 |
| 94 | 24.10 | 236.67 | 21.88 | 340.97 | 20.52 | 301.31 | 21.36 | 334.57 |
| 100 | 25.40 | 216.67 | 24.00 | 353.25 | 22.46 | 322.66 | 23.46 | 350.38 |
| 120 | 29.72 | 216.00 | 31.53 | 376.47 | 29.82 | 368.36 | 31.08 | 380.70 |
| 130 | 31.43 | 171.00 | 35.52 | 398.91 | 34.00 | 417.59 | 35.18 | 409.87 |
| 150 | 42.13 | 535.00 | 43.83 | 415.58 | 43.12 | 456.19 | 43.78 | 430.22 |
| 160 | 49.19 | 706.00 | 48.11 | 427.94 | 47.94 | 481.63 | 48.21 | 443.34 |
| 180 | 56.55 | 368.00 | 56.82 | 435.38 | 57.67 | 486.66 | 57.17 | 447.78 |
| 190 | 62.28 | 573.00 | 61.21 | 439.28 | 62.42 | 474.44 | 61.63 | 445.89 |
| 200 | 66.88 | 460.00 | 65.61 | 439.76 | 66.98 | 455.67 | 66.04 | 440.86 |
| 210 | 70.13 | 325.00 | 70.00 | 438.82 | 71.28 | 430.36 | 70.37 | 433.38 |
| 220 | 75.09 | 496.00 | 74.36 | 436.59 | 75.28 | 400.06 | 74.61 | 423.77 |
| 230 | 79.26 | 417.00 | 78.70 | 433.19 | 78.94 | 366.45 | 78.73 | 412.35 |
| 240 | 81.72 | 246.00 | 82.98 | 428.76 | 82.26 | 331.16 | 82.73 | 399.45 |
| 250 | 86.80 | 508.00 | 87.22 | 423.40 | 85.21 | 295.64 | 86.58 | 385.35 |
