# Supplementary material for: Transcriptome Analysis of Liangshan Pig Muscle Development at the Growth Curve Inflection Point and Asymptotic Stages Using Digital Gene Expression Profiling
Source: PLoS One. 2015 Aug 20;10(8):e0135978. doi: 10.1371/journal.pone.0135978 (PMC4546367; doi:10.1371/journal.pone.0135978)
Supplement: S2 Table — A is the maximum size. k is the inherent relative growth rate at the start. B is the growth curve line constant. R2 is degree of fitting. IPD: Inflection point day; IPW: Inflection point weight; IPG: Inflection point daily gain. (DOCX) [file pone.0135978.s009.docx]

**Table S2 The parameters of three growth curve models**

| **Model** | **A** | **B** | **k** | **R^2^** | **IPD(day)** | **IPW(kg)** | **IPG (g)** |
| --- | --- | --- | --- | --- | --- | --- | --- |
| Von Bertalanffy | 211.322 | 0.824 | 0.005 | 0.9971 | 193.4 | 62.61 | 429.69 |
| Logistic | 102.898 | 23.920 | 0.019 | 0.9947 | 167.1 | 51.45 | 455.43 |
| Gompertz | 146.119 | 4.212 | 0.008 | 0.9968 | 172.4 | 53.75 | 402.07 |

A is the maximum size. k is the inherent relative growth rate at the start. B is the growth curve line constant. R2 is degree of fitting. IPD: Inflection point day; IPW: Inflection point weight; IPG: Inflection point daily gain.
