# Supplementary material for: Transcriptome Analysis of Liangshan Pig Muscle Development at the Growth Curve Inflection Point and Asymptotic Stages Using Digital Gene Expression Profiling
Source: PLoS One. 2015 Aug 20;10(8):e0135978. doi: 10.1371/journal.pone.0135978 (PMC4546367; doi:10.1371/journal.pone.0135978)
Supplement: S3 Table — (DOCX) [file pone.0135978.s010.docx]

**Table S3 The information of transcriptome sequencing sample of Liangshan pigs**

| **No** | **Group** | **Weight（kg）** | **Age** | **Mean weight（kg）** |
| --- | --- | --- | --- | --- |
| L-1 | BIP | 31.20 | 143 | 31.40 |
| L-2 | BIP | 32.60 | 143 |  |
| L-3 | BIP | 30.40 | 143 |  |
| L-4 | UIP | 64.00 | 193 | 62.27 |
| L-5 | UIP | 60.20 | 193 |  |
| L-6 | UIP | 62.60 | 193 |  |
| L-7 | AIP | 89.90 | 243 | 90.90 |
| L-8 | AIP | 92.30 | 243 |  |
| L-9 | AIP | 90.50 | 243 |  |

BIP: before inflection point；UIP: under inflection point；AIP: after inflection point.
