# Supplementary material for: Transcriptome Analysis of Liangshan Pig Muscle Development at the Growth Curve Inflection Point and Asymptotic Stages Using Digital Gene Expression Profiling
Source: PLoS One. 2015 Aug 20;10(8):e0135978. doi: 10.1371/journal.pone.0135978 (PMC4546367; doi:10.1371/journal.pone.0135978)
Supplement: S4 Table — Phases 1, 4 and 9 were also used for transcriptome analysis. Phases 2, 3, 5, 6, 7, 8 and 10 were used for carcass traits and meat quality traits measured. (DOCX) [file pone.0135978.s011.docx]

| **Table S4 Information of Liangshan pigs being slaughtered** | | | |
| --- | --- | --- | --- |
| **Stages** | **Number** | **Age** | **Slaughter weight reality (kg)** |
| 1 | 3 | 143 | 31.40±1.11 |
| 2 | 3 | 165 | 53.78±2.24 |
| 3 | 3 | 175 | 58.72±2.62 |
| 4 | 3 | 193 | 62.27±1.92 |
| 5 | 3 | 205 | 69.54±2.41 |
| 6 | 3 | 215 | 74.74±1.32 |
| 7 | 3 | 225 | 80.12±1.53 |
| 8 | 3 | 235 | 85.36±1.69 |
| 9 | 3 | 243 | 90.90±1.25 |
| 10 | 3 | 255 | 95.63±2.15 |

Phases 1, 4 and 9 were also used for transcriptome analysis. Phases 2, 3, 5, 6, 7, 8 and 10 were

used for carcass traits and meat quality traits measured.
