# Supplementary material for: Transcriptome Analysis of Liangshan Pig Muscle Development at the Growth Curve Inflection Point and Asymptotic Stages Using Digital Gene Expression Profiling
Source: PLoS One. 2015 Aug 20;10(8):e0135978. doi: 10.1371/journal.pone.0135978 (PMC4546367; doi:10.1371/journal.pone.0135978)
Supplement: S5 Table — pH1 and L1 measured at 45 min postmortem; pH2 and L2 measured at 24 h postmortem. S.e. standard error. NS, no significant difference, P > 0.05; * significant at the 5% level; **significant at the 1% level; ***significant at the 0.1% level. (DOCX) [file pone.0135978.s012.docx]

**Table S5. The meat quality traits of Liangshan pig in different stages**

| **Meat traits** | **Slaughter ages** | | | | | | | | | | **S.E.** | **Level of significance** |
| --- | --- | --- | --- | --- | --- | --- | --- | --- | --- | --- | --- | --- |
|  | **1** | **2** | **3** | **4** | **5** | **6** | **7** | **8** | **9** | **10** |  |  |
| pH_1_ | 6.32 | 6.39 | 6.34 | 6.58 | 6.59 | 6.63 | 6.56 | 6.63 | 6.68 | 6.73 | 0.04 | ** |
| pH_2_ | 5.82 | 5.89 | 6.04 | 6.13 | 6.19 | 6.09 | 6.11 | 6.15 | 6.13 | 6.18 | 0.07 | * |
| L_1_ | 44.34 | 42.34 | 42.12 | 40.54 | 40.56 | 40.46 | 39.45 | 38.14 | 38.58 | 38.43 | 1.32 | * |
| L_2_ | 46.42 | 44.56 | 45.76 | 43.32 | 43.78 | 44.41 | 43.74 | 42.83 | 41.62 | 41.51 | 1.45 | * |
| Drip loss(%) | 5.22 | 5.15 | 4.65 | 4.52 | 4.63 | 4.42 | 4.28 | 4.1 | 3.96 | 4.14 | 1.03 | * |
| Marbling score | 2.25 | 2.25 | 3.33 | 3.33 | 3.67 | 3.5 | 3.33 | 3.67 | 3.87 | 3.87 | 0.86 | * |
| Share force(Kg) | 3.35 | 3.54 | 4.13 | 4.25 | 4.76 | 4.27 | 4.52 | 4.78 | 4.67 | 4.54 | 0.64 | * |
| Intramuscular fat content (%) | 2.95 | 3.43 | 3.64 | 3.53 | 4.15 | 4.38 | 4.53 | 4.65 | 4.96 | 4.97 | 0.73 | ** |
| Myofiber area(µm^2^) | 1964 | 2056 | 2275 | 2696 | 3186 | 3297 | 3386 | 3492 | 3573 | 3758 | 213 | ** |
| Cooking loss(%) | 34.32 | 34.12 | 34.63 | 33.38 | 36.95 | 35.36 | 34.72 | 35.61 | 34.74 | 35.62 | 0.74 | NS |

pH_1_ and L_1_ measured at 45 min postmortem; pH_2_ and L_2_ measured at 24 h postmortem. S.e. standard error. NS, no significant difference, *P* > 0.05;

* significant at the 5% level; **significant at the 1% level; ***significant at the 0.1% level.
