# Supplementary material for: Transcriptome Analysis of Liangshan Pig Muscle Development at the Growth Curve Inflection Point and Asymptotic Stages Using Digital Gene Expression Profiling
Source: PLoS One. 2015 Aug 20;10(8):e0135978. doi: 10.1371/journal.pone.0135978 (PMC4546367; doi:10.1371/journal.pone.0135978)
Supplement: S6 Table — Data was shown as the percentage of fatty acid to fresh weight. SFA = saturated fatty acid; UFA = unsaturated fatty acid; TFAC = total unsaturated fatty acid. S.e. standard error. NS, no significant difference, P > 0.05; * significant at the 5% level; **significant at the 1% level; ***significant at the 0.1% level. (DOCX) [file pone.0135978.s013.docx]

**Table S6. The fatty acid composition of Liangshan pig’s *longissimus dorsi* in different development stages**

| **Fatty acid (g∙100g-1)** | **BIP** | **（%）** | **UIP** | **（%）** | **AIP** | **（%）** | **S.E.** | **significance** |
| --- | --- | --- | --- | --- | --- | --- | --- | --- |
| C10:0 | 0.87 | 0.06 | 2.07 | 0.10 | 2.93 | 0.12 | 0.11 | * |
| C12:0 | 0.00 | 0.00 | 1.15 | 0.06 | 1.27 | 0.05 | 0.08 | * |
| C14:0 | 20.24 | 1.36 | 36.92 | 1.83 | 39.66 | 1.62 | 1.17 | * |
| C15:0 | 1.01 | 0.07 | 1.30 | 0.06 | 1.64 | 0.07 | 0.04 | NS |
| C16:0 | 365.50 | 24.48 | 455.39 | 22.55 | 571.40 | 23.30 | 11.47 | * |
| C17:0 | 3.84 | 0.26 | 5.21 | 0.26 | 6.18 | 0.25 | 0.13 | * |
| C18:0 | 254.64 | 17.05 | 319.75 | 15.84 | 387.74 | 15.81 | 7.40 | * |
| C20:0 | 2.89 | 0.19 | 4.88 | 0.24 | 5.44 | 0.22 | 0.15 | * |
| C21:0 | 0.96 | 0.06 | 1.04 | 0.05 | 1.34 | 0.05 | 0.02 | NS |
| C22:0 | 0.70 | 0.05 | 1.35 | 0.07 | 1.10 | 0.05 | 0.04 | * |
| C23:0 | 0.51 | 0.03 | 0.66 | 0.03 | 0.60 | 0.02 | 0.01 | NS |
| C24:0 | 0.84 | 0.06 | 1.34 | 0.07 | 1.11 | 0.05 | 0.03 | NS |
| C14:1 | 1.64 | 0.11 | 4.67 | 0.23 | 4.71 | 0.19 | 0.20 | ** |
| C15:1 | 0.57 | 0.04 | 0.67 | 0.03 | 1.21 | 0.05 | 0.04 | * |
| C16:1(9) | 29.96 | 2.01 | 65.56 | 3.25 | 79.84 | 3.26 | 2.85 | ** |
| C17:1 | 2.71 | 0.18 | 4.12 | 0.20 | 5.18 | 0.21 | 0.14 | * |
| C18:1 | 359.55 | 24.08 | 495.85 | 24.56 | 551.92 | 22.51 | 10.99 | ** |
| C20:1 | 7.80 | 0.52 | 14.07 | 0.70 | 13.21 | 0.54 | 0.38 | * |
| C22:1 | 0.76 | 0.05 | 1.11 | 0.05 | 0.87 | 0.04 | 0.02 | NS |
| C18:2 | 309.22 | 20.71 | 416.34 | 20.62 | 494.39 | 20.16 | 10.33 | * |
| C18:3n6 | 2.56 | 0.17 | 2.42 | 0.12 | 3.48 | 0.14 | 0.06 | * |
| C18:3n3 | 5.52 | 0.37 | 8.35 | 0.41 | 10.45 | 0.43 | 0.27 | * |
| C20:2 | 9.22 | 0.62 | 13.22 | 0.65 | 17.54 | 0.72 | 0.46 | ** |
| C20:3 | 2.73 | 0.18 | 3.38 | 0.17 | 4.31 | 0.18 | 0.09 | * |
| C20:4 | 101.24 | 6.78 | 150.43 | 7.45 | 232.07 | 9.46 | 7.34 | ** |
| C20:5 | 2.84 | 0.19 | 2.38 | 0.12 | 4.60 | 0.19 | 0.13 | * |
| C22:2 | 2.37 | 0.16 | 3.37 | 0.17 | 3.97 | 0.16 | 0.09 | * |
| C22:6 | 2.46 | 0.16 | 2.13 | 0.11 | 3.77 | 0.15 | 0.10 | * |
| TFA | 1493.13 |  | 2019.11 |  | 2451.95 |  | 53.35 | ** |
| SFA | 652.00 | 43.67 | 831.05 | 41.16 | 1020.42 | 41.62 | 20.47 | ** |
| MUFA | 402.98 | 26.99 | 586.05 | 29.03 | 656.94 | 26.79 | 14.56 | * |
| PUFA | 438.15 | 29.34 | 602.01 | 29.82 | 774.59 | 31.59 | 18.69 | * |
| P:S | 67.20 |  | 72.44 |  | 75.91 |  | 0.49 | * |

Data was shown as the percentage of fatty acid to fresh weight. SFA=saturated fatty acid; UFA=unsaturated fatty acid; TFAC=total unsaturated fatty acid. S.e. standard error. NS, no significant difference, *P* > 0.05; * significant at the 5% level; **significant at the 1% level; ***significant at the 0.1% level.
