# Supplementary material for: Transcriptome Analysis of Liangshan Pig Muscle Development at the Growth Curve Inflection Point and Asymptotic Stages Using Digital Gene Expression Profiling
Source: PLoS One. 2015 Aug 20;10(8):e0135978. doi: 10.1371/journal.pone.0135978 (PMC4546367; doi:10.1371/journal.pone.0135978)
Supplement: S7 Table — Data was shown as the percentage of amino acid to fresh weight and LD at 10th rib. EAA = essential amino acid; TAA = total amino acid; EAA/TAA = the ratio of EAA to TAA. a,b,c Means within a row with different superscripts indicate significant differences; S.e. standard error. NS, no significant difference, P > 0.05; * significant at the 5% level; **significant at the 1% level; ***significant at the 0.1% level. (DOCX) [file pone.0135978.s014.docx]

**Table S7. The amino acid composition of Liangshan pig’s *longissimus dorsi* in different development stages**

| **Free amino acid (mg/100g)** | **BIP** | **UIP** | **AIP** | **S.E.** | **significance** |
| --- | --- | --- | --- | --- | --- |
| Essential amino acids |  |  |  |  |  |
| Isoleucine | 2.33 | 2.62 | 2.47 | 0.02 | NS |
| Leucine | 3.86 | 4.07 | 4.02 | 0.01 | NS |
| Lysine | 2.31 | 4.55 | 2.72 | 0.13 | * |
| Threonine | 2.47 | 3.01 | 2.73 | 0.03 | * |
| Valine | 3.29 | 3.97 | 3.57 | 0.04 | NS |
| Tryptophane | 0.14 | 0.57 | 0.21 | 0.03 | * |
| Methionine | 2.42 | 1.61 | 2.49 | 0.05 | * |
| Phenylalanine | 2.96 | 2.69 | 2.88 | 0.02 | NS |
| Arginine | 1.75 | 2.77 | 2.31 | 0.06 | * |
| Histidine | 2.29 | 2.96 | 2.46 | 0.04 | NS |
| Non-essential amino acids |  |  |  |  |  |
| Asparagine | 2.79 | 1.79 | 2.34 | 0.06 | * |
| Serine | 3.48 | 3.58 | 3.74 | 0.01 | NS |
| Glycine | 8.3 | 8.24 | 6.84 | 0.09 | NS |
| glutamic | 3.54 | 3.25 | 2.74 | 0.04 | NS |
| Aspartic acid | 0.41 | 0.38 | 0.32 | 0.01 | NS |
| Citrulline | 1.03 | 0.94 | 1.07 | 0.01 | NS |
| glutamine | 18.73 | 20.16 | 16.64 | 0.20 | * |
| Tyrosine | 2.39 | 2.32 | 1.57 | 0.05 | * |
| β-alanine | 5.66 | 6.06 | 5.08 | 0.05 | * |
| Alanine | 18.69 | 22.17 | 15.29 | 0.38 | ** |
| ornithine | 0.37 | 0.48 | 0.41 | 0.01 | NS |
| TEAA | 23.82 | 28.81 | 25.85 | 0.28 | NS |
| TNAA | 65.39 | 69.39 | 56.04 | 0.76 | * |
| TFAA | 89.21 | 98.17 | 81.89 | 0.91 | * |

Data was shown as the percentage of amino acid to fresh weight and LD at 10th rib. EAA=essential amino acid; TAA=total amino acid; EAA/TAA=the ratio of EAA to TAA. a,b,c Means within a row with different superscripts indicate significant differences; S.e. standard error. *significant at the 5% level;
