# Supplementary material for: Transcriptome Analysis of Liangshan Pig Muscle Development at the Growth Curve Inflection Point and Asymptotic Stages Using Digital Gene Expression Profiling
Source: PLoS One. 2015 Aug 20;10(8):e0135978. doi: 10.1371/journal.pone.0135978 (PMC4546367; doi:10.1371/journal.pone.0135978)
Supplement: S9 Table — *: ACTB (β actin), TBP (TATA box binding protein), TOP2B (topoisomerase II β) and GCG (glucagon) are the endogenous control genes. $: Primer sequences used for DNA copy number Q-PCR. (DOCX) [file pone.0135978.s016.docx]

**Table S9. Primer sequences used for Q-PCR**

| **Target genes** | **Sequences of primers (5′→3′)** | **Products length** | **GeneBank accession No.** |
| --- | --- | --- | --- |
| *LMCD1* | F: GTGCCTTGTCTGAGATGCAA | 130 | NM_001008692 |
|  | R: TTTTCCGATCATCTTCCAGG |  |  |
| *HSP70* | F: ATGTCCGCTGCAAGAGAAGT | 216 | NM_001123127 |
|  | R: GGCGTCAAACACGGTATTCT |  |  |
| *GPI* | F: ACTGGATGGACCAGCACTTC | 159 | NM_008155 |
|  | R: GCTGGAAGTAGGCAGCAAAG |  |  |
| *TXNIP* | F: TTCTGCAATACTCTGGCGTG | 118 | NM_001044614 |
|  | R: CCATCTCATTCTCCCCTGAA |  |  |
| *CYTB* | F: CACACATCCAAACAACGAGG | 138 | NM_000101 |
|  | R: TAGTTGGCCGATGATGATGA |  |  |
| *POLR2F* | F: AGATGACTTGGAAAATGCCG | 123 | NM_001301131 |
|  | R: GCTCGCTCGTACTTGGTCAT |  |  |
| *GCG^$^* | F: GAATCAACACCATCGGTCAAAT | 147 | ENSSSCG00000015895 |
|  | R: ACCAAGCTGGTGGAGCGACCA |  |  |
| *ATP6^$^* | F: TATTTGCCTCTTTCATTGCCC | 123 | ENSSSCG00000018081 |
|  | R: GGATCGAGATTGTGCGGTTAT |  |  |
| *COX1^$^* | F: ACTACTGACAGACCGCAACC | 220 | ENSSSCG00000018075 |
|  | R: TCCAATGGACATTATGGCTC |  |  |
| *ND1^$^* | F: AGCCACATCCTCAATCTCC | 205 | ENSSSCG00000018065 |
|  | R: CCCGATGAGTGCGTATTTT |  |  |
| *ACTB* * | F: TCTGGCACCACACCTTCT | 114 | DQ178122 |
|  | R: TGATCTGGGTCATCTTCTCAC |  |  |
| *TBP* * | F: GATGGACGTTCGGTTTAGG | 124 | DQ178129 |
|  | R: AGCAGCACAGTACGAGCAA |  |  |
| *TOP2B* * | F: AACTGGATGATGCTAATGATGCT | 137 | AF222921 |
|  | R: TGGAAAAACTCCGTATCTGTCTC |  |  |

*: *ACTB* (β actin), *TBP* (TATA box binding protein), *TOP2B* (topoisomerase II β) and *GCG* (glucagon) are the endogenous control genes. $: Primer sequences used for DNA copy number Q-PCR.
